# Exploring the association between cardiovascular health and bowel health

**DOI:** 10.1038/s41598-024-62715-7

**Published:** 2024-05-23

**Authors:** Ziqing Yu, Mingyue Guo, Xiaoyin Bai, Gechong Ruan, Yinghao Sun, Wei Han, Hong Yang

**Affiliations:** 1 Department of Gastroenterology, Peking Union Medical College Hospital, Chinese Academy of Medical Sciences & Peking Union Medical College, Beijing, 100730 China; 2 Department of Epidemiology and Biostatistics, Peking Union Medical College Hospital, Chinese Academy of Medical Sciences & Peking Union Medical College, Beijing, 100730 China

**Keywords:** Cardiovascular health, Life’s essential 8, Bowel health, Chronic diarrhea, Chronic constipation, Fecal incontinence, Cardiology, Gastroenterology, Gastrointestinal diseases, Disease prevention, Public health, Epidemiology, Epidemiology

## Abstract

Chronic constipation, diarrhea, and fecal incontinence have high incidence, potential disability, and socioeconomic impact, imposing a heavy burden on the quality of life. We aim to explore the association between cardiovascular health (CVH) and bowel health from National Health and Nutrition Survey 2005–2010. CVH is assessed using Life’s Essential 8 (LE8). Chronic constipation, chronic diarrhea, and fecal incontinence are assessed based on Bristol Stool Form Scale classification, bowel movements, and bowel leakage. Better health behaviors (odds ratio [OR]: 0.71, 95% confidence interval [CI] 0.53–0.94, p = 0.02) and worse health factors (OR: 1.45, CI 1.03–2.04, p = 0.04) were associated with less chronic constipation. Less chronic diarrhea is correlated with better CVH (OR: 0.53, 95% CI 0.35–0.79, p = 0.003) and health factors (OR: 0.61, CI 0.46–0.81, p = 0.001). Meanwhile, the proportion of chronic diarrhea significantly decreases when the health behaviors score exceeds 59.42. Lower fecal incontinence was associated with better health behaviors (OR: 0.63, CI 0.44–0.90, p = 0.01) CVH. Better CVH and health behaviors are both linked to lower all-cause mortality in participants with chronic constipation and chronic diarrhea. A higher health behaviors score is also associated with less all-cause mortality in patients with fecal incontinence. Maintaining CVH at the population level contributes to intestinal health, achieving the dual management of both while saving on healthcare costs. However, further prospective research is needed to confirm these associations.

## Introduction

Functional gastrointestinal diseases, due to their high prevalence, potential disability, and significant socioeconomic impact, consume a large amount of global healthcare resources^1^. These diseases manifest as chronic diarrhea (CD), chronic constipation (CC), abdominal pain, and irritable bowel syndrome, exerting a substantial impact on public health^2^. CC and CD are common gastrointestinal disorders in the general population, with an estimated prevalence in Asia ranging from 6.1 to 28%^3,4^. CD affects approximately 1–5% of the adult population around the world, while CC has an even broader impact, with a global adult prevalence of around 16%^5,6^. In certain populations, such as elderly individuals in Finnish hospitals, the prevalence of CC can be as high as 79%^5,6^. Prolonged CD and CC can compromise the immune system, significantly affecting health and quality of life. Fecal incontinence (FI), defined as the unintentional passage of solid or liquid stool, is no longer classified as "functional" according to the Rome IV criteria^7^. The estimated prevalence of FI in non-hospitalized adults in the United States is 8.3%, with approximately 2.7% of patients experiencing incontinence symptoms at least once a week^8^. FI imposes a substantial burden on the quality of life, leading to issues such as embarrassment, social isolation, and unemployment, making it a common reason for referral to nursing homes^9,10^.

Many studies have revealed associations between CC, CD, and the cardiovascular system. A nested case–control study revealed an increased risk of CC in patients with angina and myocardial infarction^11^. In the U.S. Veterans cohort, constipation and laxative use were independently associated with the increasing risk of coronary heart disease and ischemic stroke events^12^. Another large cross-sectional study in the United States found a positive correlation between CD, constipation, and cardiovascular diseases (CVD)^13^. CVD are also common comorbidities in elderly patients with CD^14^. FI is also prevalent in stroke patients, especially in patients with hemorrhagic strokes and severe strokes^15^. CC, diarrhea, and FI also impact mortality. Lower bowel frequency has been found to be associated with cardiovascular mortality in the Japanese population^16^. CC or diarrhea is positively correlated with overall mortality and cardiovascular mortality rates^12,13^. Higher mortality and admission rates are observed in elderly community-dwelling patients with FI^17^. A study involving 41,932 participants confirmed a significant association between FI and survival rates, with an increased risk of death associated with a higher frequency of incontinence^18^. The renin-angiotensin system and the gut microbiota may mediate the interaction between gastrointestinal diseases and CVD. The angiotensin-converting enzyme/angiotensin 1–7 axis regulates immune responses, influences the composition of the microbiota, and consequently leads to gastrointestinal disorders such as diarrhea^19^. Angiotensin-converting enzyme inhibitors have also been found to induce visceral vascular edema, resulting in gastrointestinal symptoms^20^. Metabolites and microbiota in the intestines, such as trimethylamine-N-oxide, may also play an intermediary role between the heart and intestines^21^.

Since numerous studies have identified associations between CD, CC, FI, and CVD risk and mortality, we have reason to speculate that improving cardiovascular health (CVH) may have an impact on bowel health. Evaluating the relationship between CVH and bowel health can provide better guidance for population-level health management from the perspectives of public health and disease prevention. Both Life's Simple 7 and Life’s Essential 8 (LE8) are CVH indices proposed by the American Heart Association based on lifestyle factors. In comparison to LS7, LE8 incorporates sleep duration as an indicator, upgrades the scoring algorithm, and is more sensitive to individual differences^22^. The dietary quality and various food components have been shown to be closely associated with bowel health^23–26^. This may be caused by specific components in food such as oleic acid. Unsaturated fatty acids can counteract neuronal damage and reduce the concentration of low-density lipoprotein and the overall degree of systemic fat oxidation^23^. Other lifestyle factors such as unhealthy sleep pattern and smoking were also closely associated with bowel health^27–30^. Poor sleep habits can disrupt the circadian rhythm of gastrointestinal physiology, increase nocturnal awakening frequency, alter rapid eye movement sleep stages, and affect the levels of inflammatory markers, thereby impacting gut motility and susceptibility to disease^28^. Blood pressure, blood lipids, and blood glucose have also been found to be associated with CVH, and medication use may mediate this relationship^31,32^. Considering that the protective effect of CVH against all-cause mortality in the general population has been confirmed in various large-scale cohort studies, we aim to investigate the impact of CVH on all-cause mortality in individuals with CC, CD, and FI^33–36^. Assessing the impact of CVH on all-cause mortality in specific populations helps to develop more targeted preventive and healthcare strategies for chronic disease patients. Addressing the gaps in previous research, our aim is to utilize data from the National Health and Nutrition Survey (NHANES) to explore the potential association between CVH and CC, CD, and FI in the general non-institutionalized population in the United States. Additionally, we seek to examine the impact of CVH on mortality in participants with abnormal bowel health. Through this investigation, we hope to establish a foundation for the joint management of CVH and bowel health, with the goal of conserving national healthcare resources.

## Materials and methods

### Study design and population

**Figure 1:**
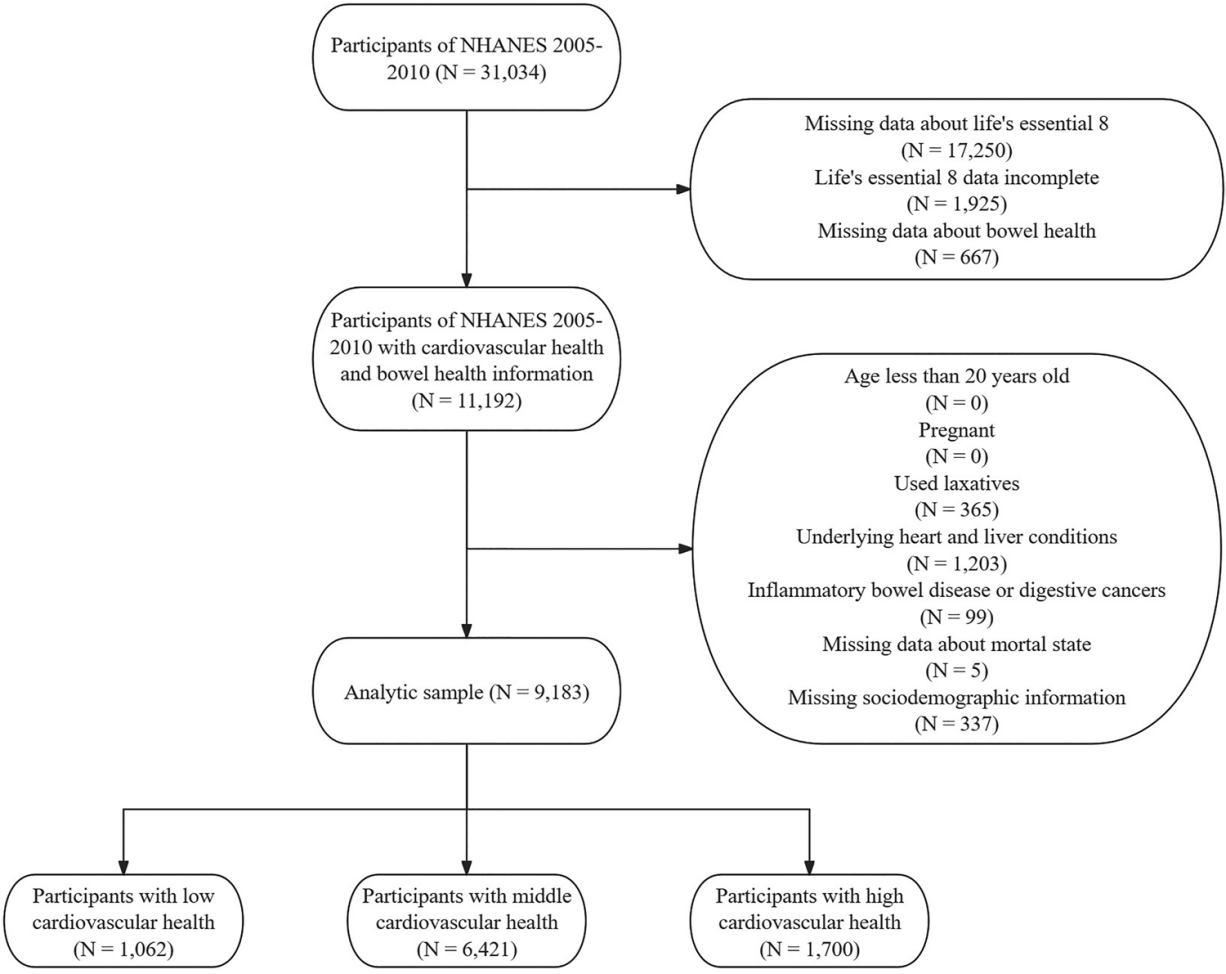
Flow chart of participants' selection and composition.

NHANES is a national survey conducted by the Centers for Disease Control and Prevention in the United States every 2 years, aimed at monitoring the public health status of the country. This survey employs a complex and rigorous sampling method, specifically a complex, stratified, multistage clustering sampling. This method facilitates the recruitment of participants, ensuring that the estimates obtained accurately represent the non-institutionalized U.S. population. NHANES covers a wide range of data, including demographic data, physical examinations, and laboratory specimens. Data collection methods include in-home interviews and visits to mobile examination centers. Trained interviewers conducted questionnaire surveys at participants' homes, while non-English/non-Spanish speakers were assisted by interpreters during home visits. Laboratory tests, physical examinations, and some questionnaire surveys were conducted at the Mobile Examination Center, ensuring controlled conditions for physical measurements at each measurement site. Additionally, some participants were recruited for post- data collection activities, including dietary telephone follow-up and home urine collection, etc. All data and guidelines are openly provided by the National Center for Health Statistics and can be accessed at https://www.cdc.gov/nchs/nhanes/index.htm. This study followed the STROBE checklist and data for this study are entirely derived from the NHANES public database, patient personal information is replaced by identification codes ("sequence"). Additionally, research for each NHANES cycle has received approval from the National Center for Health Statistics Research Ethics Review Board. Ethical approval documents are available on the official website (https://www.cdc.gov/nchs/nhanes/irba98.htm?s_cid=qr2022). All participants had provided written informed consent. We ultimately included data from three cycles (2005–2010), totaling 6 years, as only in these three year-cycles had information on bowel health. All participants from the years 2005 to 2010 were included in this study. Participants were excluded if they had missing or incomplete LE8 data (N = 19,175), missing bowel health data (N = 667), were pregnant (N = 0), age less than 20 years old (N = 0), used laxatives (N = 365), lacked follow-up data (N = 5), had missing covariates (N = 337), or had underlying heart, liver, and intestinal conditions (N = 1302, including heart failure, coronary heart disease, angina pectoris, heart attack, stroke, liver condition, inflammatory bowel disease, and cancer, but not risk factors for CVD such as diabetes, hypertension, and hyperlipidemia, Fig. 1). We ultimately included 9183 patients for analysis, representing a non-institutionalized general population of 137,712,453 in the United States.

### CVH and bowel health

CVH was evaluated by LE8, which integrates eight basic factors: diet, physical activity, smoking/nicotine exposure, sleep duration, body mass index (BMI), non-high-density lipoprotein cholesterol level, blood sugar, and blood pressure. Among them, diet, physical activity, smoking/nicotine exposure, and sleep duration constitute health behaviors, while BMI, non-high-density lipoprotein cholesterol level, blood sugar, and blood pressure constitute health factors. The score range for each indicator is from 0 to 100 points, and the LE8 total score is the unweighted average of the eight indicators^37^. The 24 diet indicators are measured using the 2015 Healthy Eating Index calculated from the 24-h dietary recall questionnaire^38^. Physical activity, nicotine exposure, sleep, diabetes history, and medication history are collected through self-reported questionnaires. Height, weight, and blood pressure, as well as non-high-density lipoprotein cholesterol, plasma glucose, and hemoglobin A1c, are obtained at the Mobile Examination Center. BMI is calculated as weight (in kg) divided by the square of height (in m). The average blood pressure is calculated from all readings recorded during the initial assessment. Serum cholesterol is measured using an enzymatic method, with non-high-density lipoprotein cholesterol being the difference between total cholesterol and high-density lipoprotein cholesterol. LE8, health behaviors, and health factors are categorized as High when the score is 80–100 points, Moderate when the score is 50–79 points, and Low when the score is 0–49 points^39^.

Intestinal health was assessed through the Bowel Health Questionnaire in the mobile exam center, utilizing an interviewer-administered Computer Assisted Personal Interviewing system. This questionnaire covered FI, fecal characteristics, and bowel movement frequency. FI was evaluated based on gas, mucus, liquid stool, and solid stool. Stool consistency was assessed using the Bristol Stool Form Scale (BSFS), along with specific questions about bowel movement frequency (How many times per week do you usually have a bowel movement?). We defined CC as BSFS Type 1 (separate hard lumps, like nuts) or Type 2 (sausage-like but lumpy) or fewer than three bowel movements per week, and CD as BSFS Types 6 (fluffy pieces with ragged edges, a mushy stool) or 7 (watery, no solid pieces) or 21 or more bowel movements per week^13,29,40–43^. BSFS Types 3, 4, 5, and other bowel movement frequency were defined as no bowel symptoms. Simultaneously, estimating intestinal health based on both stool frequency and consistency was considered superior to either alone^44^. FI was determined by the presence of bowel leakage consisting of mucus, liquid, or solid stool. If none of these occurred, it was defined as not having FI^8,45^.

### Covariates and mortal state

We conducted a comprehensive analysis incorporating a wide range of sociodemographic characteristics using NHANES extensive questionnaire and laboratory data. Age and age group (20–40, 40–60, > 60) were utilized as continuous and categorical variables in subsequent analyses, respectively. Gender is divided into male and female, while ethnicity is categorized as white, black, and other. Additionally, family size (1–3, > 3), annual family income (under $20,000, $20,000–$35,000, $35,000–$75,000, over $75,000), smoking status (never, former, now), drinking status (never, former, now), and alcohol consumption (grams per day) were also included. BMI was categorized as underweight/normal weight (< 25 kg/m^2^), overweight (25–29.9 kg/m^2^), and obese (≥ 30 kg/m^2^). The diagnosis of diabetes followed the Global guideline for type 2 diabetes, or it was based on information from a doctor, or the use of diabetes-related medications.^46^ High blood pressure was defined as individuals taking antihypertensive medication, those informed of having hypertension by a doctor, or those with a systolic blood pressure ≥ 130 mmHg or diastolic blood pressure ≥ 80 mmHg^47^. Depression was assessed using the 9-question Patient Health Questionnaire (PHQ-9) and categorized into five groups based on PHQ-9 scores (none, mild, moderate, moderately severe, and severe depression from scores of 0–4, 5–9, 10–14, 15–19, and 20–27)^48^. The mortality status and causes of death for each NHANES participant were determined by matching with death certificate records from the National Death Index (https://www.cdc.gov/nchs/data-linkage/mortality-public.htm). All-cause mortality served as the follow-up endpoint for this study, with the follow-up period ending in December 2019.

### Statistical analysis

Considering the complexity of the NHANES sampling survey, we selected appropriate weights for sample analysis to better reflect the characteristics of the general non-institutionalized population in the United States. Descriptive statistical analysis was employed to present the basic characteristics of participants (continuous variables presented as weighted mean ± standard error, categorical variables presented as weighted numbers and percentages). The independent student t test, ANOVA, and Chi-square test were used to test demographic differences among participants. Multiple models, including various covariates, were chosen to better assess the association between CVH and bowel health (Supplementary Methods). Briefly, Model 1 to 3 was adjusted for covariates to varying degrees. Among them, Model 3 represents the final model after fully adjustment. Based on cutoff points of 50 and 80, the LE8 score, health behavior score, and health factor score were divided into three categories (Low, Moderate, High) for segmented analysis. Weighted logistic regression was similarly employed to test the association between CVH and bowel health. For sensitivity analysis, we followed the approach outlined by Busgang et al., excluding participants who used gastrointestinal medications (e.g., antidiarrheals, laxatives, etc.) and psychotherapeutic medications (i.e., antidepressants, anticonvulsants, and antipsychotics).^49^ Subsequently, we conducted multivariable analysis again. Additionally, we analyzed the impact of CVH on the mortality of individuals suffering from CC, CD, and FI. Restrictive cubic splines were used to further explore potential nonlinear associations between CVH and bowel health. All statistical analyses and graphical representations were conducted using R-4.2.2.

### Ethics declarations

Since the data for this study are entirely derived from the NHANES public database, patient personal information is replaced by identification codes ("sequence"), thus obviating the need for ethical approval from the authors' institution. Additionally, as stated in the Methods section, research for each NHANES cycle has received approval from the National Center for Health Statistics Research Ethics Review Board. Ethical approval documents are available on the official website (https://www.cdc.gov/nchs/nhanes/irba98.htm?s_cid=qr2022). All participants had provided written informed consent.

## Results

### Characteristics of the study participants

Table 1 displays the basic sociodemographic characteristics of participants classified by CVH. Among the 9183 participants, 1062 (11.56%) had low CVH, 6421 (69.92%) had moderate CVH, and 1700 (18.51%) had high CVH, representing approximately 137,712,453 general non-institutionalized individuals in the United States. The average age of all participants was 45.79, with females accounting for 52.57%, and a predominant representation of White (73.94%). Comparing participants with low CVH, participants with high CVH were younger, had a higher proportion of females, more White individuals, fewer individuals living alone, higher educational levels, higher family incomes, lower rates of smoking and alcohol consumption, healthier body weight status, more frequent vigorous exercise, and greater milk consumption. Regarding comorbidities, individuals with high CVH had lower prevalence rates of diabetes, hyperlipidemia, hypertension, and milder degrees of depression (Table 2). For bowel health, participants with high CVH experienced fewer cases of CC, CD, and FI (Table 2).

**Table 1:** Baseline sociodemographic characteristics of participants by CVH.

Variable	Total	Low (n = 1062)	Moderate (n = 6421)	High (n = 1700)	P value
Age	45.79 (0.35)	50.64 (0.69)	46.62 (0.34)	40.96 (0.66)	< 0.0001
Age group					< 0.0001
20–40	52,742,731.07 (38.30)	3,164,055.18 (24.41)	34,405,630.58 (36.12)	15,173,045.31 (51.43)	
40–60	55,883,333.51 (40.58)	5,922,076.10 (45.68)	39,498,162.07 (41.47)	10,463,095.33 (35.47)	
≥ 60	29,086,389.90 (21.12)	3,877,311.56 (29.91)	21,345,296.67 (22.41)	3,863,781.67 (13.10)	
Sex					< 0.0001
Female	72,389,894.68 (52.57)	6,710,218.96 (51.76)	47,235,769.91 (49.59)	18,443,905.80 (62.52)	
Male	65,322,559.81 (47.43)	6,253,223.88 (48.24)	48,013,319.41 (50.41)	11,056,016.52 (37.48)	
Ethnicity					< 0.0001
White	101,827,747.32 (73.94)	8,720,746.36 (67.27)	70,146,852.74 (73.65)	22,960,148.22 (77.83)	
Black	13,649,802.57 (9.91)	2,337,857.74 (18.03)	9,752,045.68 (10.24)	1,559,899.16 (5.29)	
Other	22,234,904.59 (16.15)	1,904,838.75 (14.69)	15,350,190.90 (16.12)	4,979,874.94 (16.88)	
Marital status					0.003
Married/with partner	90,272,062.89 (65.55)	7,630,000.36 (58.86)	63,656,941.74 (66.83)	18,985,120.79 (64.36)	
Alone	47,440,391.59 (34.45)	5,333,442.48 (41.14)	31,592,147.58 (33.17)	10,514,801.53 (35.64)	
Educational level					< 0.0001
≤ Highschool	54,522,738.40 (39.59)	7,791,630.90 (60.10)	40,642,109.04 (42.67)	6,088,998.46 (20.64)	
> Highschool	83,189,716.09 (60.41)	5,171,811.95 (39.90)	54,606,980.28 (57.33)	23,410,923.86 (79.36)	
Family size					0.53
1–3	94,791,565.70 (68.83)	9,207,514.10 (71.03)	65,215,924.42 (68.47)	2,0368,127.18 (69.04)	
> 3	42,920,888.78 (31.17)	3,755,928.74 (28.97)	30,033,164.90 (31.53)	9,131,795.14 (30.96)	
Annual family income					< 0.0001
Under $20,000	21,878,448.66 (15.89)	3,473,591.92 (26.80)	14,914,256.18 (15.66)	3,490,600.56 (11.83)	
$20,000–$35,000	27,197,950.44 (19.75)	3,558,045.08 (27.45)	19,050,760.83 (20.00)	4,589,144.53 (15.56)	
$35,000–$75,000	43,165,008.43 (31.34)	3,610,119.87 (27.85)	31,936,742.01 (33.53)	7,618,146.55 (25.82)	
Over $75,000	45,471,046.95 (33.02)	2,321,685.97 (17.91)	29,347,330.30 (30.81)	13,802,030.68 (46.79)	
Smoking status					< 0.0001
Never	74,599,785.95 (54.17)	2,983,007.69 (23.01)	47,947,669.57 (50.34)	23,669,108.70 (80.23)	
Former	33,198,707.95 (24.11)	3,086,589.59 (23.81)	24,846,696.09 (26.09)	5,265,422.27 (17.85)	
Now	29,913,960.58 (21.72)	6,893,845.56 (53.18)	22,454,723.66 (23.57)	565,391.35 (1.92)	
Alcohol consumption (g/day)	9.62 (0.34)	8.74 (1.01)	9.78 (0.44)	9.49 (0.62)	0.62
Drinking status					< 0.0001
Never	14,008,575.66 (10.17)	1,175,175.63 (9.07)	8,984,832.71 (9.43)	3,848,567.31 (13.05)	
Former	21,057,471.91 (15.29)	3,547,931.56 (27.37)	15,157,965.70 (15.91)	2,351,574.64 (7.97)	
Now	102,646,406.92 (74.54)	8,240,335.65 (63.57)	711,062,90.91 (74.65)	23,299,780.37 (78.98)	
BMI	28.47 (0.15)	33.85 (0.32)	29.04 (0.13)	24.24 (0.14)	< 0.0001
Weight status					< 0.0001
Under/normal weight	44,592,813.96 (32.38)	941,861.11 (7.27)	24,772,148.72 (26.01)	18,878,804.13 (64.00)	
Overweight	46,609,118.83 (33.85)	2,626,672.73 (20.26)	35,248,573.54 (37.01)	8,733,872.56 (29.61)	
Obese	46,510,521.70 (33.77)	9,394,909.01 (72.47)	35,228,367.06 (36.99)	1,887,245.63 (6.40)	
Vigorous physical activity					< 0.0001
No	81,494, ,311.32 (59.18)	10,691,378.90 (82.47)	59,460,191.47 (62.43)	11,342,740.95 (38.45)	
Yes	56,218,143.16 (40.82)	2,272,063.94 (17.53)	35,788,897.85 (37.57)	18,157,181.37 (61.55)	
Milk consumption					0.002
Never	20,298,081.71 (14.74)	2,454,058.86 (18.93)	14,400,694.84 (15.12)	3,443,328.01 (11.67)	
Rarely	18,958,069.49 (13.77)	1,970,232.09 (15.20)	13,468,186.64 (14.14)	3,519,650.76 (11.93)	
Sometimes	39,870,572.44 (28.95)	3,593,638.55 (27.72)	27,772,194.53 (29.16)	8,504,739.36 (28.83)	
Often	58,585,730.85 (42.54)	4,945,513.34 (38.15)	39,608,013.31 (41.58)	14,032,204.20 (47.57)	

CVH: cardiovascular health; BMI: Body Mass Index.

Low was defined as a LE8 score of 0 to 49, moderate of 50–79, and high of 80–100.

Continuous variables were analyzed using independent Student's t-test or ANOVA, while categorical variables were assessed using the Chi-square test. A significance level of p < 0.05 was considered statistically significant. All data, except for the numbers next to the titles, represent results that have been weighted.

**Table 2:** Baseline health characteristics of participants by CVH.

Variable	Total	Low (n = 1062)	Moderate (n = 6421)	High (n = 1700)	P value
Depression					< 0.0001
None	108,291,728.25 (78.64)	8,260,043.66 (63.72)	74,055,996.82 (77.75)	25,975,687.78 (88.05)	
Mild	20,486,369.25 (14.88)	2,539,500.38 (19.59)	15,073,851.09 (15.83)	2,873,017.78 (9.74)	
Moderate	5,982,262.87 (4.34)	1,230,503.39 (9.49)	4,333,504.67 (4.55)	418,254.81 (1.42)	
Moderately severe	2,399,750.85 (1.74)	644,363.56 (4.97)	1,522,425.34 (1.60)	232,961.95 (0.79)	
Severe	552,343.26 (0.40)	289,031.85 (2.23)	263,311.41 (0.28)	0.00 (0.00)	
DM					< 0.0001
No	114,371,736.98 (83.05)	8,035,849.03 (61.99)	78,938,039.81 (82.88)	27,397,848.14 (92.87)	
IFG/IGT	11,325,103.62 (8.22)	1,346,456.96 (10.39)	8,423,667.32 (8.84)	1,554,979.34 (5.27)	
DM	12,015,613.88 (8.73)	3,581,136.85 (27.62)	7,887,382.18 (8.28)	547,094.84 (1.85)	
Hyperlipidemia					< 0.0001
No	38,680,784.94 (28.09)	783,725.38 (6.05)	22,210,864.77 (23.32)	15,686,194.79 (53.17)	
Yes	99,031,669.54 (71.91)	12,179,717.46 (93.95)	73,038,224.55 (76.68)	13,813,727.53 (46.83)	
Hypertension					< 0.0001
No	91,921,858.19 (66.75)	4,689,270.41 (36.17)	60,692,329.05 (63.72)	26,540,258.73 (89.97)	
Yes	45,790,596.29 (33.25)	8,274,172.43 (63.83)	34,556,760.27 (36.28)	2,959,663.59 (10.03)	
LE8 score	68.34 (0.37)	42.36 (0.18)	66.19 (0.17)	86.67 (0.20)	< 0.0001
Health behaviors score	65.17 (0.54)	37.02 (0.76)	63.16 (0.37)	84.02 (0.42)	< 0.0001
HEI-2015 diet score	38.88 (0.81)	18.27 (1.12)	34.94 (0.75)	60.63 (1.12)	< 0.0001
Physical activity score	69.53 (0.86)	29.05 (1.56)	68.49 (0.89)	90.68 (0.80)	< 0.0001
Nicotine exposure score	69.27 (0.70)	36.09 (1.73)	66.71 (0.64)	92.10 (0.86)	< 0.0001
Sleep health score	83.01 (0.49)	64.68 (1.17)	82.50 (0.53)	92.69 (0.52)	< 0.0001
Health factors score	71.51 (0.37)	47.70 (0.59)	69.23 ( (0.28)	89.32 (0.37)	< 0.0001
Body mass index score	63.30 (0.72)	35.11 (1.46)	59.97 (0.62)	86.46 (0.68)	< 0.0001
Blood lipids score	61.86 (0.47)	38.75 (1.25)	58.64 (0.51)	82.40 (0.80)	< 0.0001
Blood glucose score	89.31 (0.32)	68.48 (1.22)	89.32 (0.36)	98.43 (0.30)	< 0.0001
Blood pressure score	71.55 (0.56)	48.44 (1.28)	68.99 (0.62)	90.00 (0.67)	< 0.0001
Health behaviors classification					< 0.0001
Low	28,817,053.27 (20.93)	9,897,101.86 (76.35)	18,919,951.41 (19.86)	0.00 (0.00)	
Moderate	69,434,482.00 (50.42)	3,038,575.09 (23.44)	57,760,683.27 (60.64)	8,635,223.64 (29.27)	
High	39,460,919.21 (28.65)	27,765.89 (0.21)	18,568,454.65 (19.49)	20,864,698.68 (70.73)	
Health factors classification					< 0.0001
Low	18,023,982.98 (13.09)	7,389,588.53 (57.00)	10,634,394.45 (11.16)	0.00 (0.00)	
Moderate	68,820,596.88 (49.97)	5,313,805.62 (40.99)	58,349,112.45 (61.26)	5,157,678.80 (17.48)	
High	50,867,874.63 (36.94)	260,048.69 (2.01)	26,265,582.42 (27.58)	24,342,243.52 (82.52)	
Bowel health					< 0.0001
Normal	110,494,562.27 (80.24)	9,446,152.34 (72.87)	76,089,504.86 (79.88)	24,958,905.07 (84.61)	
Chronic constipation	12,767,411.76 (9.27)	1,737,667.84 (13.40)	8,254,662.18 (8.67)	2,775,081.74 (9.41)	
Chronic diarrhea	14,450,480.45 (10.49)	1,779,622.66 (13.73)	10,904,922.28 (11.45)	1,765,935.51 (5.99)	
Fecal incontinence					< 0.0001
No	127,287,164.47 (92.43)	11,536,521.98 (88.99)	87,705,618.18 (92.08)	28,045,024.30 (95.07)	
Yes	10,425,290.01 (7.57)	1,426,920.86 (11.01)	7,543,471.14 (7.92)	1,454,898.02 (4.93)	
Follow-up (person-months)	140.25 (0.78)	135.07 (1.69)	140.86 (0.84)	140.58 (1.19)	0.01
Mortal status					< 0.0001
Assumed alive	125,613,977.79 (91.21)	10,546,461.11 (81.36)	86,519,902.93 (90.84)	28,547,613.75 (96.77)	
Assumed deceased	12,098,476.70 (8.79)	2,416,981.73 (18.64)	8,729,186.39 (9.16)	952,308.57 (3.23)	

CVH: cardiovascular health; LE8: life’s essential 8; HEI: healthy eating index; DM: Diabetes mellitus; IGT; impaired glucose tolerance; IFG: Impaired Fasting Glucose.

Low was defined as a LE8 score of 0 to 49, moderate of 50–79, and high of 80–100.

Continuous variables were analyzed using independent Student’s t-test or ANOVA, while categorical variables were assessed using the Chi-square test. A significance level of p < 0.05 was considered statistically significant. All data, except for the numbers next to the titles, represent results that have been weighted.

Supplementary Tables 1 and 2 presents the basic characteristics of participants classified based on health behaviors and health factors. Participants with high health behaviors and high health factors exhibited higher proportions of CC, CD, and FI.

### Association between CVH and bowel health

Table 3 illustrates the associations between CVH, health behaviors, health factors, and bowel health. After fully adjusted, the multivariate analysis revealed that better CVH was associated with fewer instances of CD (odds ratio [OR]: 0.53, 95% confidence interval [CI] 0.35–0.79, p = 0.003). Although CC (OR: 0.81, CI 0.56–1.17, p = 0.26) and FI (OR: 0.74, CI 0.51–1.07, p = 0.11) were not significantly correlated with CVH, the odds ratios remained less than one. Additionally, a high level of health behaviors was linked to a reduced occurrence of CC (OR: 0.71, CI 0.53–0.94, p = 0.02), while high health factors were associated with a lower prevalence of CD (OR: 0.61, CI 0.46–0.81, p = 0.001). It’s worth noting that the high health factor is positively correlated with the prevalence of CC (OR: 1.45, CI 1.03–2.04, p = 0.04). A separate analysis of each component of health factors in relation to CC was conducted. We found that only the grading of BMI scores showed a positive correlation with the proportion of CC (OR: 1.78, CI 1.37–2.30, p < 0.001). Multifactorial analysis focusing on BMI and CC further confirmed this observation (Supplementary Table 3).

**Table 3:** The association between bowel health, fecal incontinence and CVH.

	LE8 classification							
	Crude model		Model 1		Model 2		Model 3	
	95% CI	P	95% CI	P	95% CI	P	95% CI	P
Chronic constipation								
Low	Ref		Ref		Ref			
Moderate	0.61 (0.46, 0.82)	0.002	0.58 (0.44, 0.77)	< 0.001	0.73 (0.55, 0.98)	0.04		
High	0.67 (0.48, 0.94)	0.02	0.50 (0.36, 0.71)	< 0.001	0.81 (0.56, 1.17)	0.26		
P for trend		0.13		0.002		0.48		
Chronic diarrhea								
Low	Ref		Ref		Ref			
Moderate	0.81 (0.63, 1.04)	0.10	0.83 (0.65, 1.06)	0.13	0.91 (0.71, 1.16)	0.44		
High	0.40 (0.28, 0.57)	< 0.0001	0.43 (0.30, 0.62)	< 0.0001	0.53 (0.35, 0.79)	0.003		
P for trend		< 0.0001		< 0.0001		< 0.001		
Fecal incontinence								
Low	Ref		Ref		Ref			
Moderate	0.70 (0.53, 0.91)	0.01	0.78 (0.60, 1.01)	0.06	0.92 (0.72, 1.18)	0.51		
High	0.42 (0.30, 0.59)	< 0.0001	0.55 (0.39, 0.77)	< 0.001	0.74 (0.51, 1.07)	0.11		
P for trend		< 0.0001		< 0.001		0.09		

	Health behaviors classification							
	95% CI	P	95% CI	P	95% CI	P	95% CI	P
Chronic constipation								
Low	Ref		Ref		Ref		Ref	
Moderate	0.70 (0.56, 0.87)	0.002	0.72 (0.59, 0.88)	0.002	0.86 (0.70, 1.04)	0.12	0.87 (0.71, 1.07)	0.19
High	0.53 (0.40, 0.70)	< 0.0001	0.52 (0.40, 0.67)	< 0.0001	0.72 (0.54, 0.95)	0.02	0.71 (0.53, 0.94)	0.02
P for trend		< 0.0001		< 0.0001		0.02		0.02
Chronic diarrhea								
Low	Ref		Ref		Ref		Ref	
Moderate	1.00 (0.76, 1.31)	1.00	0.98 (0.74, 1.28)	0.85	1.08 (0.82, 1.43)	0.58	1.06 (0.80, 1.42)	0.67
High	0.77 (0.56, 1.06)	0.10	0.74 (0.54, 1.02)	0.07	0.90 (0.62, 1.29)	0.55	0.92 (0.63, 1.33)	0.63
P for trend		0.07		0.05		0.5		0.59
Fecal incontinence								
Low	Ref		Ref		Ref		Ref	
Moderate	0.82 (0.64, 1.05)	0.11	0.75 (0.58, 0.96)	0.03	0.89 (0.68, 1.15)	0.35	0.89 (0.69, 1.15)	0.37
High	0.66 (0.48, 0.90)	0.01	0.55 (0.40, 0.75)	< 0.001	0.71 (0.49, 1.01)	0.06	0.72 (0.50, 1.05)	0.08
P for trend		0.01		< 0.001		0.05		0.08

	Health factors classification							
	95% CI	P	95% CI	P	95% CI	P	95% CI	P
Chronic constipation								
Low	Ref		Ref		Ref		Ref	
Moderate	0.91 (0.67, 1.23)	0.52	0.90 (0.65, 1.23)	0.49	1.00 (0.71, 1.40)	0.99	1.01 (0.72, 1.42)	0.96
High	1.41 (1.05, 1.87)	0.02	1.10 (0.79, 1.52)	0.58	1.42 (1.01, 1.99)	0.04	1.45 (1.03, 2.04)	0.04
P for trend		0.001		0.27		0.01		0.01
Chronic diarrhea								
Low	Ref		Ref		Ref		Ref	
Moderate	0.82 (0.64, 1.05)	0.11	0.83 (0.64, 1.07)	0.14	0.87 (0.68, 1.11)	0.25	0.87 (0.68, 1.11)	0.26
High	0.51 (0.40, 0.66)	< 0.0001	0.56 (0.42, 0.75)	< 0.001	0.61 (0.46, 0.81)	0.001	0.61 (0.46, 0.81)	0.001
P for trend		< 0.0001		< 0.001		< 0.001		< 0.001
Fecal incontinence								
Low	Ref		Ref		Ref		Ref	
Moderate	0.76 (0.55, 1.04)	0.09	0.91 (0.65, 1.26)	0.56	0.96 (0.70, 1.33)	0.81	0.97 (0.70, 1.33)	0.82
High	0.48 (0.35, 0.65)	< 0.0001	0.77 (0.55, 1.09)	0.13	0.86 (0.61, 1.22)	0.38	0.86 (0.61, 1.22)	0.39
P for trend		< 0.0001		0.12		0.36		0.37

**LE8 classification.**

Crude model: Unadjusted model.

Model 1: Adjusted for age and sex.

Model 2: Additionally adjusted for marital status, educational level, family size, annual family income, alcohol, and PHQ-9.

**Health behaviors classification.**

Crude model: Unadjusted model.

Model 1: Adjusted for age and sex.

Model 2: Additionally adjusted for marital status, educational level, family size, annual family income, alcohol, and PHQ-9.

Model 3: Additionally adjusted for, BMI, DM, Hypertension, Hyperlipidemia.

**Health factors classification.**

Crude model: Unadjusted model.

Model 1: Adjusted for age and sex.

Model 2: Additionally adjusted for marital status, educational level, family size, annual family income, alcohol, and PHQ-9.

Model 3: Additionally adjusted for vigorous physical activity and smoke.

CVH: cardiovascular health; LE8: life’s essential 8; OR: odds ratios; CI confidence interval; BMI: Body Mass Index; DM: Diabetes mellitus;

Low was defined as a score of 0 to 49, moderate of 50–79, and high of 80–100.

We stratified by age, gender, race, marital status, education level, household income, family size, smoking, alcohol consumption, BMI, depression, and physical activity, adjusting for relevant covariates. Supplementary Tables 4–12 present the results of the stratified analyses for CVH, health behaviors, and health factors concerning CC, CD, and FI. Except for the association of health behaviors with CD and health factors with FI, CVH, health behaviors, and health factors were consistently correlated with lower rates of CC, CD, and FI in specific subpopulations.

### Restricted cubic spline analysis of CVH with bowel health

**Figure 2:**
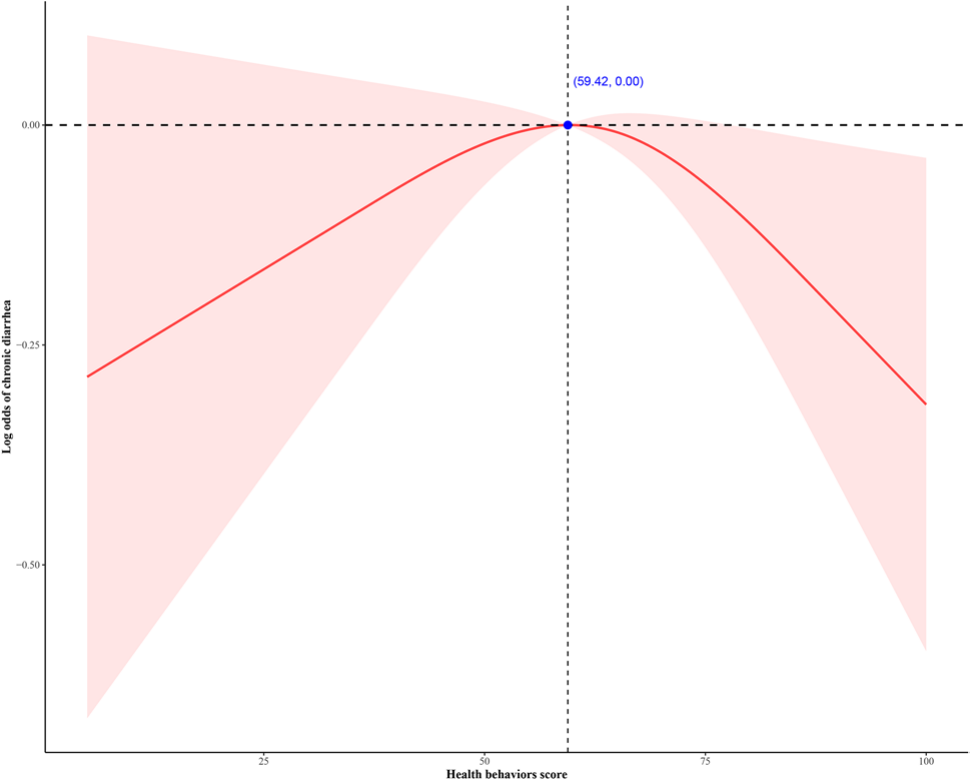
The association between chronic diarrhea and health behaviors score. An inverted U-shaped relationship is evident in the context of health behaviors and chronic diarrhea, wherein a notable reduction in the prevalence of chronic diarrhea is observed once the health behaviors score surpasses 59.42.

Figure 2 illustrates the non-linear relationship between the health behaviors score and CD. An inverted U-shaped association is observed between the health behaviors and CD, with a significant decrease in the proportion of CD when the health behaviors score exceeds 59.42.

### Association between CVH and all-cause mortality in patients with abnormal bowel health

Table 4 illustrates the associations between CVH and all-cause mortality in participants with CC, CD, and FI. After adjusting for all confounding variables, a better CVH was correlated with a lower all-cause mortality rate in patients with CC (OR: 0.19, CI 0.08–0.43, p < 0.001) and CD (OR: 0.30, CI 0.11–0.83, p = 0.02). High health behaviors were associated with a lower all-cause mortality in participants with CC (OR: 0.24, CI 0.13–0.44, p < 0.0001), CD (OR: 0.48, CI 0.24–0.96, p = 0.04), and FI (OR: 0.59, CI 0.35–0.98, p = 0.04). However, we did not observe an impact of health factors on the mortality in participants with poorer intestinal health (Table 4).

**Table 4:** The association between CVH and death of patients with chronic constipation, chronic diarrhea and fecal incontinence.

	LE8 classification							
	Crude model		Model 1		Model 2		Model 3	
	95% CI	P	95% CI	P	95% CI	P	95% CI	P
Chronic constipation								
Low	Ref		Ref		Ref			
Moderate	0.41 (0.22, 0.78)	0.01	0.49 (0.26, 0.91)	0.02	0.53 (0.30, 0.96)	0.04		
High	0.10 (0.03, 0.30)	< 0.0001	0.16 (0.07, 0.35)	< 0.0001	0.19 (0.08, 0.43)	< 0.0001		
P for trend		< 0.0001		< 0.0001		< 0.0001		
Chronic diarrhea								
Low	Ref		Ref		Ref			
Moderate	0.61 (0.38, 0.98)	0.04	0.65 (0.37, 1.15)	0.14	0.81 (0.44, 1.48)	0.49		
High	0.13 (0.05, 0.30)	< 0.0001	0.19 (0.07, 0.51)	< 0.001	0.30 (0.11, 0.83)	0.02		
P for trend		< 0.0001		0.01		0.12		
Fecal incontinence								
Low	Ref		Ref		Ref			
Moderate	0.88 (0.54, 1.43)	0.60	0.67 (0.40, 1.10)	0.11	0.82 (0.50, 1.34)	0.43		
High	0.44 (0.22, 0.90)	0.02	0.38 (0.18, 0.83)	0.01	0.70 (0.36, 1.37)	0.30		
P for trend		0.02		0.01		0.3		

Character	Health behaviors classification							
	Crude model		Model 1		Model 2		Model 3	
	95% CI	P	95% CI	P	95% CI	P	95% CI	P
Chronic constipation								
Low	Ref		Ref		Ref		Ref	
Moderate	0.68 (0.40, 1.19)	0.18	0.51 (0.31, 0.83)	0.01	0.56 (0.34, 0.92)	0.02	0.61 (0.35, 1.03)	0.07
High	0.55 (0.31, 0.96)	0.04	0.25 (0.15, 0.43)	< 0.0001	0.28 (0.16, 0.50)	< 0.0001	0.24 (0.13, 0.44)	< 0.0001
P for trend		0.05		< 0.0001		< 0.0001		< 0.0001
Chronic diarrhea								
Low	Ref		Ref		Ref		Ref	
Moderate	0.87 (0.50, 1.50)	0.61	0.70 (0.40, 1.20)	0.19	0.86 (0.48, 1.51)	0.59	0.88 (0.47, 1.63)	0.68
High	0.49 (0.24, 0.99)	0.048	0.32 (0.17, 0.62)	< 0.001	0.48 (0.26, 0.90)	0.02	0.48 (0.24, 0.96)	0.04
P for trend		0.05		< 0.001		0.03		0.05
Fecal incontinence								
Low	Ref		Ref		Ref		Ref	
Moderate	0.74 (0.46, 1.20)	0.23	0.53 (0.32, 0.87)	0.01	0.67 (0.44, 1.02)	0.06	0.63 (0.40, 1.00)	0.049
High	0.76 (0.43, 1.36)	0.35	0.38 (0.21, 0.68)	0.001	0.56 (0.36, 0.87)	0.01	0.59 (0.35, 0.98)	0.04
P for trend		0.34		0.002		0.01		0.05

Character	Health factors classification							
	Crude model		Model 1		Model 2		Model 3	
	95% CI	P	95% CI	P	95% CI	P	95% CI	P
Chronic constipation								
Low	Ref		Ref		Ref		Ref	
Moderate	0.62 (0.40, 0.97)	0.04	0.92 (0.61, 1.38)	0.68	0.90 (0.56, 1.45)	0.67	0.96 (0.58, 1.58)	0.87
High	0.23 (0.12, 0.43)	< 0.0001	0.77 (0.44, 1.34)	0.36	0.85 (0.50, 1.48)	0.57	0.89 (0.50, 1.58)	0.69
P for trend		< 0.0001		0.35		0.57		0.68
Chronic diarrhea								
Low	Ref		Ref		Ref		Ref	
Moderate	0.90 (0.51, 1.59)	0.71	0.91 (0.46, 1.83)	0.80	0.99 (0.52, 1.91)	0.98	0.89 (0.45, 1.74)	0.72
High	0.43 (0.20, 0.92)	0.03	0.88 (0.37, 2.08)	0.77	0.95 (0.39, 2.27)	0.90	0.83 (0.34, 2.04)	0.68
P for trend		0.02		0.77		0.91		0.69
Fecal incontinence								
Low	Ref		Ref		Ref		Ref	
Moderate	0.90 (0.51, 1.59)	0.72	0.94 (0.55, 1.63)	0.84	1.08 (0.65, 1.81)	0.76	0.97 (0.55, 1.72)	0.92
High	0.59 (0.34, 1.02)	0.06	1.19 (0.70, 2.04)	0.52	1.61 (0.89, 2.91)	0.12	1.30 (0.69, 2.43)	0.41
P for trend		0.05		0.58		0.16		0.47

**LE8 classification.**

Crude model: Unadjusted model.

Model 1: Adjusted for age and sex.

Model 2: Additionally adjusted for marital status, educational level, family size, annual family income, alcohol, and PHQ-9.

**Health behaviors classification.**

Crude model: Unadjusted model.

Model 1: Adjusted for age and sex.

Model 2: Additionally adjusted for marital status, educational level, family size, annual family income, alcohol, and PHQ-9.

Model 3: Additionally adjusted for, BMI, DM, Hypertension, Hyperlipidemia.

**Health factors classification.**

Crude model: Unadjusted model.

Model 1: Adjusted for age and sex.

Model 2: Additionally adjusted for marital status, educational level, family size, annual family income, alcohol, and PHQ-9.

Model 3: Additionally adjusted for vigorous physical activity and smoke.

CVH: cardiovascular health; LE8: life’s essential 8; OR: odds ratios; CI confidence interval; BMI: Body Mass Index; DM: Diabetes mellitus;

Low was defined as a score of 0 to 49, moderate of 50–79, and high of 80–100.

Supplementary Tables 13–21 present the results of stratified analyses for CVH, health behaviors, and health factors on all-cause mortality in participants with CC, CD, and FI. Better CVH, health behaviors, and health factors were observed to be associated with lower all-cause mortality in certain subgroups.

### Restricted cubic spline analysis of CVH and all-cause mortality in patients with abnormal bowel health

**Figure 3:**
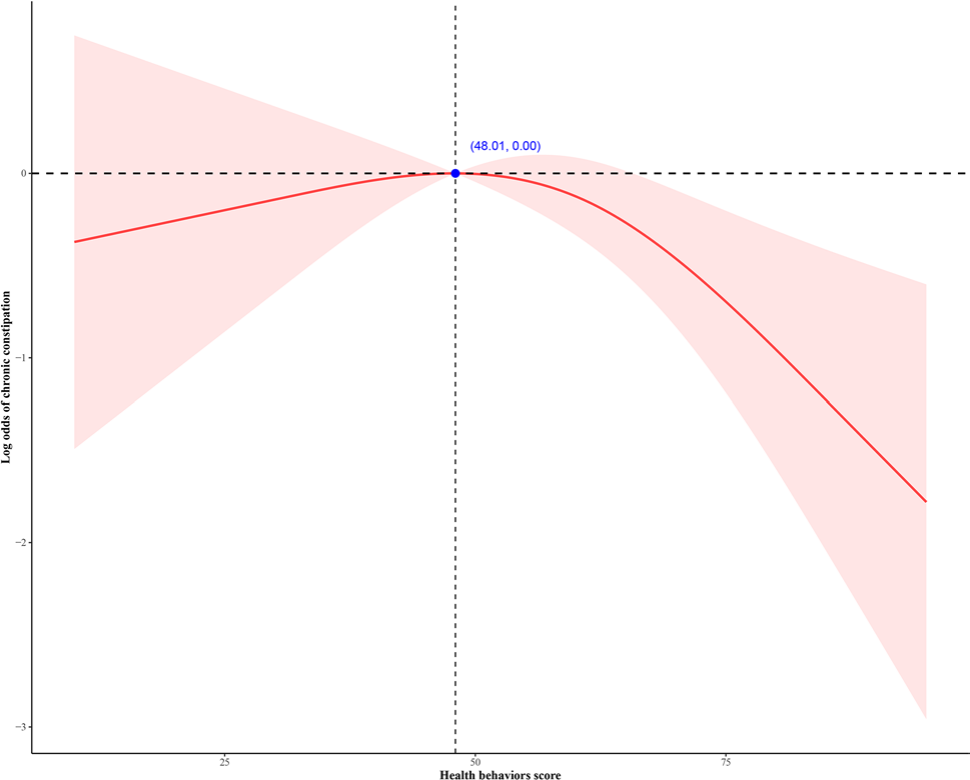
The association between all-cause mortality and health behaviors in participants with chronic constipation. The health behaviors score exhibits an inverted J-shaped correlation with all-cause mortality in CC patients. Beyond health behaviors score of 48.01, there is a decrease in the mortality rate among CC patients with an increase in the health behaviors score.

Figure 3 illustrates the non-linear relationship between health behaviors and all-cause mortality in participants with CC. The health behaviors score demonstrates an inverted J-shaped relationship with all-cause mortality in CC patients. When the health behaviors score exceeds 48.01, the mortality rate in CC patients decreases with an increase in the health behaviors score.

### Sensitivity analysis

After excluding participants using gastrointestinal and psychotherapeutic medications, the relationship between CVH and bowel health remained consistent with the main analysis (Supplementary Table 22). Additionally, better health behaviors were significantly associated with lower FI (OR: 0.63, CI 0.44–0.90, p = 0.01), while only a trend was observed in the main analysis.

## Discussion

This large cross-sectional study discovered that lower proportion of CC was associated with higher health behaviors score and lower health factors score. Better CVH and health factors were associated with lower rates of CD. Moreover, there was a significant decrease in the proportion of CD when the health behaviors score exceeded 59.42. FI showed no association with CVH, but with health behaviors. Better CVH and health behaviors were both correlated with lower all-cause mortality in participants with CC and CD. Better health behaviors was also associated with lower all-cause mortality in patients with FI.

Many previous studies have found an association between bowel health (CC, CD, and FI) and CVD^11,12,14,43^. The common influencing factors for both (such as diet, exercise, sleep, nicotine exposure) have played a certain role. High saturated fat intake and low dietary fiber intake are significantly associated with an increased rate of constipation^23,24^. And high intake of lycopene, α-carotene, and phosphorus may reduce the risk of CC^50,51^. A healthy dietary pattern, including a higher intake of viscous soluble fibers, can lower cholesterol to promote CVH. It can also normalize stool consistency in patients with constipation and diarrhea, and improve the blood sugar, lipid levels, and blood pressure^52^. A large cross-sectional study in the United States found that participants engaged in moderate or vigorous recreational activities had a lower risk of constipation^53^. Both shorter and longer sleep durations are associated with an increased risk of constipation^27,28^. CD is also closely related to sleep quality^54^. Exposure to environmental tobacco increases the risk of CC, especially in individuals with poor dietary quality^29^. Using electronic cigarettes may also lead to varying degrees of gastrointestinal symptoms, such as vomiting and diarrhea^30^. Adopting healthy lifestyle such as quitting smoking, maintaining a balanced diet, regular exercise, and healthy sleep duration contributes to achieving better intestinal health.

Health factors, especially body mass index, have different impacts on CD or FI compared to CC. Overweight or obesity is positively correlated with cardiovascular events and FI or CD^55,56^. We found BMI is positively correlated with CD, and negatively correlated with CC. In two studies incorporating representative populations, an increased risk of CD was observed with rising BMI, along with a higher frequency of daily bowel movements^57,58^. While the relationship between the risk of CC and increased BMI is not significant, a reduction in the daily frequency of bowel movements is significantly associated with BMI^58^. Further large-scale prospective studies are still needed to explore the potential connections between BMI and bowel health in the general population. The prevalence of CD and FI significantly increases in diabetes patients, and hypertension and hyperlipidemia are also significantly associated with CC^31,32,59^. Micronutrients, such as magnesium, may be an important factor linking high blood sugar, and hypertension with CD. Serum magnesium levels play a crucial role in the pathophysiological mechanisms of prehypertension, with high blood sugar being a significant cause of hypomagnesemia^60–62^. Meanwhile, CD is also a significant cause of hypomagnesemia^63^. Furthermore, Bile acids, as a central factor in the gut-liver axis, establish a close connection between lipid metabolism and intestinal health^64^.

The renin-angiotensin system and the gut microbiota may serve as a link between gut health and CVH. Studies suggest that the angiotensin converting enzyme/angiotensin 1–7 axis can modulate immune responses, influence the composition of the microbiota through factors like neural stimuli, body weight, fat, and lipid levels, and thereby lead to a connection between CVD and the gut^19^. Angiotensin converting enzyme inhibitor have also been reported to induce visceral angioedema, leading to gastrointestinal symptoms^20^. Many cases have been reported where CD appeared after taking angiotensin type 1 receptor blockers^65^. Microbiota and gut metabolites such as trimethylamine-*N*-oxide may act as intermediaries in the relationship between the heart and the gut^21^. The complex interplay between bowel health and CVH requires further researches for confirmation.

The impact of CVH on mortality has been confirmed in many studies^33–36^. In a broader general population, the protective effect of CVH on mortality has been observed, especially in individuals with a higher polygenic risk score for CVD^43,66,67^. We observed that BETTER CVH, especially the health behavior, is associated with lower all-cause mortality in patients with CC, CD, and FI. This may suggest that health behaviors can improve the prognosis of patients with abnormal bowel health. Physical exercise and CVH can increase the perceived benefits for patients^68^. The association between CVH and prognosis may be due to the negative correlation between CVH and physiological age and phenotypic age acceleration, where oxidative stress plays a crucial mediating role^39,69^.

The design of cross-sectional studies only explores the association between CVH and bowel health, providing limited estimation regarding their causal relationship and direction of effect. We also could not observe the impact of dynamic CVH changes on intestinal health. Furthermore, our study predominantly focuses on white individuals, and the results may not be applicable to populations of other specific races or countries. It is important to note that our findings cannot be extrapolated to the entire U.S. population, as part of the NHANES data do not cover all participants. Despite these limitations, our study possesses several strengths. To our knowledge, this is the first study assessing the relationship between CVH and bowel health in the general U.S. population and all-cause mortality in individuals with abnormal intestinal health. We included only participants with complete eight indicators, ensuring the accuracy of CVH calculation and the reliability of the results. We excluded participants taking laxatives and those with underlying diseases such as cardiovascular, liver, and gastrointestinal conditions to better evaluate the association between CVH and intestinal health in the general population.

## Conclusions

In conclusion, we found that high CVH is associated with a lower proportion of CD, as well as lower all-cause mortality in patients with CC and CD. No association was observed between FI and CVH, but healthy behaviors can improve the prognosis in patients with FI. Maintaining good CVH at the population level contributes to bowel health, suggesting a potential connection between cardiovascular and bowel health. However, prospective studies are needed to further confirm these findings.

### Supplementary Information

Supplementary Information.

## Data Availability

The datasets generated and/or analysed during the current study are available in the NHANES repository, https://www.cdc.gov/nchs/nhanes/index.htm.

## Abbreviations

NHANES: National Health and Nutrition Survey
CD: Chronic diarrhea
CC: Chronic constipation
FI: Fecal incontinence
CVD: Cardiovascular diseases
CVH: Cardiovascular health
LE8: Life’s Essential 8
BMI: Body mass index
PHQ-9: 9-Question Patient Health Questionnaire;
BSFS: Bristol Stool Form Scale
CI: 95% Confidence interval
OR: Odds ratio
