# Supplementary material for: Exploring the association between cardiovascular health and bowel health
Source: Sci Rep. 2024 May 23;14:11819. doi: 10.1038/s41598-024-62715-7 (PMC11116406; doi:10.1038/s41598-024-62715-7)
Supplement: Supplementary file 1 — Supplementary Information. [file 41598_2024_62715_MOESM1_ESM.docx]

**Title: Exploring the association Between Cardiovascular Health and Bowel Health**

**Short Title: Cardiovascular Health and Bowel Health**

Ziqing Yu^1#^, Mingyue Guo^1#^, Xiaoyin Bai^1^, Gechong Ruan^1^, Yinghao Sun^1^, Wei Han^2^, Hong Yang^1^

^1^Department of Gastroenterology, Peking Union Medical College Hospital, Chinese Academy of Medical Sciences & Peking Union Medical College, Beijing 100730, China

^2^Department of Epidemiology and Biostatistics, Peking Union Medical College Hospital, Chinese Academy of Medical Sciences & Peking Union Medical College, Beijing 100730, China

^#^Ziqing Yu and Mingyue Guo contributed equally to this study.

Corresponding author: Hong Yang, Tel: 010-69155014, E-mail: [yangh@pumch.cn](mailto:yangh@pumch.cn)

**Caption for supplementary material**

Appendix 1. Supplementary Methods (page 2)

Appendix 2. Supplementary Tables (page 3–­25)

Appendix 1. Supplementary Methods

Specifically, when Life’s Essential 8 (LE8) was included in the analysis, closely related variables (including vigorous physical activity, smoking, body mass index (BMI), diabetes mellitus, hypertension, and hyperlipidemia) were no longer included in the model. Similarly, when health behaviors and health factors were included in the analysis, variables closely related to them were also excluded from the model (vigorous physical activity and smoking for health behaviors, and BMI, diabetes mellitus, hypertension, and hyperlipidemia for health factors). Crude model was unadjusted. For LE8, model 1 was adjusted for age and sex. Model 2 was adjusted for age, sex, marital status, educational level, family size, annual family income, alcohol, and 9-question Patient Health Questionnaire (PHQ-9). For health behaviors, model 1 was adjusted for age and sex. Model 2 was adjusted for age, sex, marital status, educational level, family size, annual family income, alcohol, and PHQ-9. Model 3 was adjusted for age, sex, marital status, educational level, family size, annual family income, alcohol, PHQ-9, BMI, diabetes, Hypertension, Hyperlipidemia. For health factors, model 1 was adjusted for age and sex. Model 2 was adjusted for age, sex, marital status, educational level, family size, annual family income, alcohol, and PHQ-9. Model 3 was adjusted for age, sex, marital status, educational level, family size, annual family income, alcohol, PHQ-9, vigorous physical activity and smoke.

**Appendix 1. Supplementary tables**

**Supplementary table 1. Baseline characteristics of participants by health behaviors**

| **Variable** | **Total** | **Low (n=2088)** | **Moderate (n=4671)** | **High (n=2424)** | **P value** |
| --- | --- | --- | --- | --- | --- |
| **Age** | 45.79(0.35) | 43.39(0.47) | 45.54(0.38) | 47.97(0.63) | < 0.0001 |
| **Sex** |  |  |  |  | 0.01 |
| **Female** | 72389894.68(52.57) | 14948748.17(51.87) | 35286477.20(50.82) | 22154669.31(56.14) |  |
| **Male** | 65322559.81(47.43) | 13868305.10(48.13) | 34148004.80(49.18) | 17306249.90(43.86) |  |
| **Ethnic** |  |  |  |  | < 0.0001 |
| **White** | 101827747.32(73.94) | 20210701.69(70.13) | 50441804.11(72.65) | 31175241.52(79.00) |  |
| **Black** | 13649802.57(9.91) | 4477967.88(15.54) | 6980926.69(10.05) | 2190908.01(5.55) |  |
| **Other** | 22234904.59(16.15) | 4128383.70(14.33) | 12011751.20(17.30) | 6094769.69(15.45) |  |
| **Marital status** |  |  |  |  | < 0.0001 |
| **Married/With partner** | 90272062.89(65.55) | 17003421.05(59.00) | 45648356.77(65.74) | 27620285.08(69.99) |  |
| **Alone** | 47440391.59(34.45) | 11813632.22(41.00) | 23786125.24(34.26) | 11840634.14(30.01) |  |
| **Educational level** |  |  |  |  | < 0.0001 |
| **≤Highschool** | 54522738.40(39.59) | 16255829.48(56.41) | 28031490.08(40.37) | 10235418.83(25.94) |  |
| **>Highschool** | 83189716.09(60.41) | 12561223.78(43.59) | 41402991.92(59.63) | 29225500.38(74.06) |  |
| **Family size** |  |  |  |  | 0.01 |
| **1-3** | 94791565.70(68.83) | 18619103.02(64.61) | 47778718.59(68.81) | 28393744.09(71.95) |  |
| **>3** | 42920888.78(31.17) | 10197950.25(35.39) | 21655763.41(31.19) | 11067175.12(28.05) |  |
| **Annual family income** |  |  |  |  | < 0.0001 |
| **Under $20,000** | 21878448.66(15.89) | 6934048.72(24.06) | 10730657.63(15.45) | 4213742.31(10.68) |  |
| **$20,000 - $35,000** | 27197950.44(19.75) | 7369867.43(25.57) | 13188679.38(18.99) | 6639403.64(16.83) |  |
| **$35,000 - $75,000** | 43165008.43(31.34) | 8564506.92(29.72) | 23622368.58(34.02) | 10978132.94(27.82) |  |
| **Over $75,000** | 45471046.95(33.02) | 5948630.20(20.64) | 21892776.42(31.53) | 17629640.33(44.68) |  |
| **Smoking status** |  |  |  |  | < 0.0001 |
| **Never** | 74599785.95(54.17) | 4238103.54(14.71) | 38566514.10(55.54) | 31795168.32(80.57) |  |
| **Former** | 33198707.95(24.11) | 5498617.09(19.08) | 20034339.97(28.85) | 7665750.89(19.43) |  |
| **Now** | 29913960.58(21.72) | 19080332.65(66.21) | 10833627.94(15.60) | 0.00(0.00) |  |
| **Alcohol consumption (g/d)** | 9.62(0.34) | 10.75(0.66) | 9.17(0.51) | 9.59(0.54) | 0.11 |
| **Drinking status** |  |  |  |  | < 0.0001 |
| **Never** | 14008575.66(10.17) | 1707764.88(5.93) | 7238298.59(10.42) | 5062512.18(12.83) |  |
| **Former** | 21057471.91(15.29) | 5947118.05(20.64) | 10654093.85(15.34) | 4456260.01(11.29) |  |
| **Now** | 102646406.92(74.54) | 21162170.34(73.44) | 51542089.56(74.23) | 29942147.02(75.88) |  |
| **BMI** | 28.47(0.15) | 28.57(0.22) | 28.93(0.18) | 27.57(0.19) | < 0.0001 |
| **Weight status** |  |  |  |  | < 0.0001 |
| **Under/Normal weight** | 44592813.96(32.38) | 9908000.73(34.38) | 20244549.40(29.16) | 14440263.82(36.59) |  |
| **Overweight** | 46609118.83(33.85) | 8814910.25(30.59) | 23949689.38(34.49) | 13844519.19(35.08) |  |
| **Obese** | 46510521.70(33.77) | 10094142.29(35.03) | 25240243.22(36.35) | 11176136.20(28.32) |  |
| **PHQ-9 (cut-off by 5)** |  |  |  |  | < 0.0001 |
| **No** | 108291728.25(78.64) | 18728620.99(64.99) | 54878841.69(79.04) | 34684265.57(87.90) |  |
| **Yes** | 29420726.23(21.36) | 10088432.28(35.01) | 14555640.31(20.96) | 4776653.64(12.10) |  |
| **PHQ-9 (cut-off by 10)** |  |  |  |  | < 0.0001 |
| **No** | 128778097.50(93.51) | 24554836.63(85.21) | 65643265.81(94.54) | 38579995.06(97.77) |  |
| **Yes** | 8934356.98(6.49) | 4262216.63(14.79) | 3791216.19(5.46) | 880924.16(2.23) |  |
| **Vigorous physical activity** |  |  |  |  | < 0.0001 |
| **No** | 81494311.32(59.18) | 21983308.76(76.29) | 41632376.09(59.96) | 17878626.47(45.31) |  |
| **Yes** | 56218143.16(40.82) | 6833744.50(23.71) | 27802105.92(40.04) | 21582292.74(54.69) |  |
| **DM** |  |  |  |  | 0.28 |
| **No** | 114371736.98(83.05) | 23642947.84(82.04) | 57432636.94(82.71) | 33296152.21(84.38) |  |
| **IFG/IGT** | 11325103.62(8.22) | 2253809.78(7.82) | 5946627.14(8.56) | 3124666.70(7.92) |  |
| **DM** | 12015613.88(8.73) | 2920295.65(10.13) | 6055217.92(8.72) | 3040100.31(7.70) |  |
| **Hyperlipidemia** |  |  |  |  | 0.02 |
| **No** | 38680784.94(28.09) | 7014669.71(24.34) | 19925578.42(28.70) | 11740536.81(29.75) |  |
| **Yes** | 99031669.54(71.91) | 21802383.56(75.66) | 49508903.58(71.30) | 27720382.40(70.25) |  |
| **Hypertension** |  |  |  |  | 0.77 |
| **No** | 91921858.19(66.75) | 18930002.02(65.69) | 46341578.98(66.74) | 26650277.19(67.54) |  |
| **Yes** | 45790596.29(33.25) | 9887051.25(34.31) | 23092903.02(33.26) | 12810642.02(32.46) |  |
| **Metabolic syndrome** |  |  |  |  | < 0.001 |
| **No** | 99002516.19(71.89) | 19568765.42(67.91) | 49437878.63(71.20) | 29995872.14(76.01) |  |
| **Yes** | 38709938.30(28.11) | 9248287.85(32.09) | 19996603.38(28.80) | 9465047.07(23.99) |  |
| **LE8 score** | 68.34(0.37) | 53.54(0.31) | 67.61(0.25) | 80.42(0.37) | < 0.0001 |
| **Health behaviors score** | 65.17(0.54) | 36.17(0.35) | 64.47(0.18) | 87.58(0.20) | < 0.0001 |
| **HEI-2015 diet score** | 38.88(0.81) | 16.57(0.65) | 32.92(0.76) | 65.65(0.76) | < 0.0001 |
| **Physical activity score** | 69.53(0.86) | 39.50(1.27) | 66.74(0.95) | 96.37(0.33) | < 0.0001 |
| **Nicotine exposure score** | 69.27(0.70) | 25.13(1.15) | 73.19(0.80) | 94.60(0.28) | < 0.0001 |
| **Sleep health score** | 83.01(0.49) | 63.50(0.78) | 85.02(0.59) | 93.70(0.32) | < 0.0001 |
| **Health factors score** | 71.51(0.37) | 70.91(0.58) | 70.76(0.40) | 73.26(0.64) | < 0.001 |
| **Body mass index score** | 63.30(0.72) | 63.23(1.12) | 60.87(0.84) | 67.63(1.05) | < 0.0001 |
| **Blood lipids score** | 61.86(0.47) | 60.00(0.95) | 62.13(0.59) | 62.73(0.93) | 0.1 |
| **Blood glucose score** | 89.31(0.32) | 87.96(0.57) | 89.20(0.33) | 90.49(0.63) | 0.01 |
| **Blood pressure score** | 71.55(0.56) | 72.46(0.99) | 70.83(0.74) | 72.17(0.94) | 0.32 |
| **LE8 classification** |  |  |  |  | < 0.0001 |
| **Low** | 12963442.84(9.41) | 9897101.86(34.34) | 3038575.09(4.38) | 27765.89(0.07) |  |
| **Moderate** | 95249089.32(69.17) | 18919951.41(65.66) | 57760683.27(83.19) | 18568454.65(47.06) |  |
| **High** | 29499922.32(21.42) | 0.00(0.00) | 8635223.64(12.44) | 20864698.68(52.87) |  |
| **Health factors classification** |  |  |  |  | 0.004 |
| **Low** | 18023982.98(13.09) | 4323247.55(15.00) | 9533130.20(13.73) | 4167605.23(10.56) |  |
| **Moderate** | 68820596.88(49.97) | 13805833.55(47.91) | 35428469.22(51.02) | 19586294.10(49.63) |  |
| **High** | 50867874.63(36.94) | 10687972.17(37.09) | 24472882.58(35.25) | 15707019.88(39.80) |  |
| **Bowel health** |  |  |  |  | < 0.0001 |
| **Normal** | 110494562.27(80.24) | 21981609.10(76.28) | 55332508.48(79.69) | 33180444.69(84.08) |  |
| **Chronic constipation** | 12767411.76(9.27) | 3616012.97(12.55) | 6350439.59(9.15) | 2800959.21(7.10) |  |
| **Chronic diarrhea** | 14450480.45(10.49) | 3219431.20(11.17) | 7751533.93(11.16) | 3479515.32(8.82) |  |
| **Fecal incontinence** |  |  |  |  | 0.02 |
| **No** | 127287164.47(92.43) | 26171491.70(90.82) | 64125355.12(92.35) | 36990317.66(93.74) |  |
| **Yes** | 10425290.01(7.57) | 2645561.57(9.18) | 5309126.89(7.65) | 2470601.56(6.26) |  |
| **Follow-up (person-months)** | 140.25(0.78) | 140.74(1.21) | 141.55(0.88) | 137.62(1.06) | 0.01 |
| **Mortal status** |  |  |  |  | < 0.001 |
| **Assumed alive** | 125613977.79(91.21) | 25497380.91(88.48) | 63276166.62(91.13) | 36840430.25(93.36) |  |
| **Assumed deceased** | 12098476.70(8.79) | 3319672.36(11.52) | 6158315.38(8.87) | 2620488.96(6.64) |  |

LE8: life’s essential 8; HEI: healthy eating index; PHQ-9: 9-question Patient Health Questionnaire; BMI: Body Mass Index; DM: Diabetes mellitus; IGT; impaired glucose tolerance; IFG: Impaired Fasting Glucose;

Low was defined as a Health behaviors score of 0 to 49, moderate of 50–79, and high of 80–100

Continuous variables were analyzed using independent Student's t-test or ANOVA, while categorical variables were assessed using the Chi-square test. A significance level of p < 0.05 was considered statistically significant. All data, except for the numbers next to the titles, represent results that have been weighted.

**Supplementary table 2. Baseline characteristics of participants by health factors**

| **Variable** | **Total** | **Low (n=1410)** | **Moderate (n=4715)** | **High (n=3058)** | **P value** |
| --- | --- | --- | --- | --- | --- |
| **Age** | 45.79(0.35) | 54.72(0.57) | 48.74(0.38) | 38.61(0.45) | < 0.0001 |
| **Sex** |  |  |  |  | < 0.0001 |
| **Female** | 72389894.68(52.57) | 9367884.92(51.97) | 32606805.15(47.38) | 30415204.61(59.79) |  |
| **Male** | 65322559.81(47.43) | 8656098.06(48.03) | 36213791.72(52.62) | 20452670.02(40.21) |  |
| **Ethnic** |  |  |  |  | < 0.001 |
| **White** | 101827747.32(73.94) | 13091323.34(72.63) | 50888237.43(73.94) | 37848186.55(74.40) |  |
| **Black** | 13649802.57(9.91) | 2460530.86(13.65) | 6703378.76(9.74) | 4485892.95(8.82) |  |
| **Other** | 22234904.59(16.15) | 2472128.78(13.72) | 11228980.68(16.32) | 8533795.13(16.78) |  |
| **Marital status** |  |  |  |  | < 0.0001 |
| **Married/With partner** | 90272062.89(65.55) | 11983726.00(66.49) | 47057630.33(68.38) | 31230706.57(61.40) |  |
| **Alone** | 47440391.59(34.45) | 6040256.98(33.51) | 21762966.55(31.62) | 19637168.06(38.60) |  |
| **Educational level** |  |  |  |  | < 0.0001 |
| **≤Highschool** | 54522738.40(39.59) | 8904118.07(49.40) | 29066214.87(42.23) | 16552405.46(32.54) |  |
| **>Highschool** | 83189716.09(60.41) | 9119864.91(50.60) | 39754382.00(57.77) | 34315469.17(67.46) |  |
| **Family size** |  |  |  |  | < 0.0001 |
| **1-3** | 94791565.70(68.83) | 13356825.74(74.11) | 48249235.11(70.11) | 33185504.85(65.24) |  |
| **>3** | 42920888.78(31.17) | 4667157.23(25.89) | 20571361.76(29.89) | 17682369.78(34.76) |  |
| **Annual family income** |  |  |  |  | < 0.001 |
| **Under $20,000** | 21878448.66(15.89) | 3534314.63(19.61) | 9937903.26(14.44) | 8406230.77(16.53) |  |
| **$20,000 - $35,000** | 27197950.44(19.75) | 3852872.38(21.38) | 13786272.58(20.03) | 9558805.49(18.79) |  |
| **$35,000 - $75,000** | 43165008.43(31.34) | 6024743.03(33.43) | 22864837.13(33.22) | 14275428.28(28.06) |  |
| **Over $75,000** | 45471046.95(33.02) | 4612052.95(25.59) | 22231583.90(32.30) | 18627410.10(36.62) |  |
| **Smoking status** |  |  |  |  | < 0.0001 |
| **Never** | 74599785.95(54.17) | 9531409.41(52.88) | 37098974.40(53.91) | 27969402.15(54.98) |  |
| **Former** | 33198707.95(24.11) | 5340485.19(29.63) | 17502876.82(25.43) | 10355345.93(20.36) |  |
| **Now** | 29913960.58(21.72) | 3152088.38(17.49) | 14218745.66(20.66) | 12543126.55(24.66) |  |
| **Alcohol consumption (g/d)** | 9.62(0.34) | 8.97(1.16) | 9.53(0.44) | 9.97(0.45) | 0.6 |
| **Drinking status** |  |  |  |  | < 0.0001 |
| **Never** | 14008575.66(10.17) | 2168944.77(12.03) | 6583931.27(9.57) | 5255699.62(10.33) |  |
| **Former** | 21057471.91(15.29) | 4436197.70(24.61) | 11368649.69(16.52) | 5252624.52(10.33) |  |
| **Now** | 102646406.92(74.54) | 11418840.51(63.35) | 50868015.92(73.91) | 40359550.49(79.34) |  |
| **BMI** | 28.47(0.15) | 35.31(0.22) | 29.94(0.15) | 24.04(0.09) | < 0.0001 |
| **Weight status** |  |  |  |  | < 0.0001 |
| **Under/Normal weight** | 44592813.96(32.38) | 228726.11(1.27) | 11207900.22(16.29) | 33156187.63(65.18) |  |
| **Overweight** | 46609118.83(33.85) | 2605621.77(14.46) | 28434387.96(41.32) | 15569109.09(30.61) |  |
| **Obese** | 46510521.70(33.77) | 15189635.10(84.27) | 29178308.69(42.40) | 2142577.90(4.21) |  |
| **PHQ-9 (cut-off by 5)** |  |  |  |  | 0.002 |
| **No** | 108291728.25(78.64) | 13276490.07(73.66) | 54121565.21(78.64) | 40893672.97(80.39) |  |
| **Yes** | 29420726.23(21.36) | 4747492.90(26.34) | 14699031.66(21.36) | 9974201.67(19.61) |  |
| **PHQ-9 (cut-off by 10)** |  |  |  |  | 0.08 |
| **No** | 128778097.50(93.51) | 16535529.14(91.74) | 64343145.41(93.49) | 47899422.95(94.16) |  |
| **Yes** | 8934356.98(6.49) | 1488453.84(8.26) | 4477451.46(6.51) | 2968451.68(5.84) |  |
| **Vigorous physical activity** |  |  |  |  | < 0.0001 |
| **No** | 81494311.32(59.18) | 13570952.22(75.29) | 42242216.77(61.38) | 25681142.33(50.49) |  |
| **Yes** | 56218143.16(40.82) | 4453030.76(24.71) | 26578380.11(38.62) | 25186732.30(49.51) |  |
| **DM** |  |  |  |  | < 0.0001 |
| **No** | 114371736.98(83.05) | 10020331.63(55.59) | 56302586.12(81.81) | 48048819.24(94.46) |  |
| **IFG/IGT** | 11325103.62(8.22) | 2032908.79(11.28) | 7129691.27(10.36) | 2162503.56(4.25) |  |
| **DM** | 12015613.88(8.73) | 5970742.56(33.13) | 5388319.49(7.83) | 656551.83(1.29) |  |
| **Hyperlipidemia** |  |  |  |  | < 0.0001 |
| **No** | 38680784.94(28.09) | 768935.03(4.27) | 10365303.00(15.06) | 27546546.91(54.15) |  |
| **Yes** | 99031669.54(71.91) | 17255047.95(95.73) | 58455293.88(84.94) | 23321327.72(45.85) |  |
| **Hypertension** |  |  |  |  | < 0.0001 |
| **No** | 91921858.19(66.75) | 3952014.36(21.93) | 41852035.56(60.81) | 46117808.27(90.66) |  |
| **Yes** | 45790596.29(33.25) | 14071968.62(78.07) | 26968561.31(39.19) | 4750066.36(9.34) |  |
| **Metabolic syndrome** |  |  |  |  | < 0.0001 |
| **No** | 99002516.19(71.89) | 4216145.05(23.39) | 45999572.58(66.84) | 48786798.56(95.91) |  |
| **Yes** | 38709938.30(28.11) | 13807837.93(76.61) | 22821024.30(33.16) | 2081076.07(4.09) |  |
| **LE8 score** | 68.34(0.37) | 51.31(0.33) | 65.51(0.32) | 78.20(0.39) | < 0.0001 |
| **Health behaviors score** | 65.17(0.54) | 62.23(0.59) | 65.52(0.60) | 65.73(0.75) | < 0.0001 |
| **HEI-2015 diet score** | 38.88(0.81) | 36.42(1.12) | 38.90(0.91) | 39.71(1.08) | 0.04 |
| **Physical activity score** | 69.53(0.86) | 60.90(1.49) | 69.79(1.05) | 72.23(1.02) | < 0.0001 |
| **Nicotine exposure score** | 69.27(0.70) | 71.51(1.21) | 70.34(0.93) | 67.02(1.29) | 0.05 |
| **Sleep health score** | 83.01(0.49) | 80.10(1.02) | 83.05(0.53) | 83.97(0.71) | 0.01 |
| **Health factors score** | 71.51(0.37) | 40.38(0.26) | 65.49(0.19) | 90.67(0.15) | < 0.0001 |
| **Body mass index score** | 63.30(0.72) | 26.71(0.72) | 54.73(0.66) | 87.87(0.49) | < 0.0001 |
| **Blood lipids score** | 61.86(0.47) | 34.65(1.17) | 52.31(0.49) | 84.41(0.50) | < 0.0001 |
| **Blood glucose score** | 89.31(0.32) | 63.30(1.13) | 89.21(0.40) | 98.67(0.21) | < 0.0001 |
| **Blood pressure score** | 71.55(0.56) | 36.85(1.01) | 65.73(0.64) | 91.73(0.50) | < 0.0001 |
| **LE8 classification** |  |  |  |  | < 0.0001 |
| **Low** | 12963442.84(9.41) | 7389588.53(41.00) | 5313805.62(7.72) | 260048.69(0.51) |  |
| **Moderate** | 95249089.32(69.17) | 10634394.45(59.00) | 58349112.45(84.78) | 26265582.42(51.63) |  |
| **High** | 29499922.32(21.42) | 0.00(0.00) | 5157678.80(7.49) | 24342243.52(47.85) |  |
| **Health behaviors classification** |  |  |  |  | 0.004 |
| **Low** | 28817053.27(20.93) | 4323247.55(23.99) | 13805833.55(20.06) | 10687972.17(21.01) |  |
| **Moderate** | 69434482.00(50.42) | 9533130.20(52.89) | 35428469.22(51.48) | 24472882.58(48.11) |  |
| **High** | 39460919.21(28.65) | 4167605.23(23.12) | 19586294.10(28.46) | 15707019.88(30.88) |  |
| **Bowel health** |  |  |  |  | < 0.0001 |
| **Normal** | 110494562.27(80.24) | 13978488.29(77.55) | 55431691.58(80.55) | 41084382.41(80.77) |  |
| **Chronic constipation** | 12767411.76(9.27) | 1533523.36(8.51) | 5354523.10(7.78) | 5879365.30(11.56) |  |
| **Chronic diarrhea** | 14450480.45(10.49) | 2511971.32(13.94) | 8034382.20(11.67) | 3904126.93(7.68) |  |
| **Fecal incontinence** |  |  |  |  | < 0.001 |
| **No** | 127287164.47(92.43) | 16094713.72(89.30) | 63074329.38(91.65) | 48118121.37(94.59) |  |
| **Yes** | 10425290.01(7.57) | 1929269.25(10.70) | 5746267.49(8.35) | 2749753.27(5.41) |  |
| **Follow-up (person-months)** | 140.25(0.78) | 135.59(1.19) | 139.34(1.02) | 143.13(0.89) | < 0.0001 |
| **Mortal status** |  |  |  |  | < 0.0001 |
| **Assumed alive** | 125613977.79(91.21) | 15168140.57(84.16) | 62041945.43(90.15) | 48403891.79(95.16) |  |
| **Assumed deceased** | 12098476.70(8.79) | 2855842.40(15.84) | 6778651.45(9.85) | 2463982.84(4.84) |  |

LE8: life’s essential 8; HEI: healthy eating index; PHQ-9: 9-question Patient Health Questionnaire; BMI: Body Mass Index; DM: Diabetes mellitus; IGT; impaired glucose tolerance; IFG: Impaired Fasting Glucose;

Low was defined as a Health factors score of 0 to 49, moderate of 50–79, and high of 80–100

Continuous variables were analyzed using independent Student's t-test or ANOVA, while categorical variables were assessed using the Chi-square test. A significance level of p < 0.05 was considered statistically significant. All data, except for the numbers next to the titles, represent results that have been weighted.

**Supplementary Table 3. The association between chronic constipation and individual scores of health factors**

| **Chronic constipation** | **BMI score classification** | | | | | | | |
| --- | --- | --- | --- | --- | --- | --- | --- | --- |
|  | crude model | | Model 1 | | Model 2 | | Model 3 | |
|  | 95%CI | P | 95%CI | P | 95%CI | P | 95%CI | P |
| Low | ref |  | ref |  | ref |  | ref |  |
| Moderate | 1.21(0.94,1.55) | 0.14 | 1.42(1.10,1.82) | 0.01 | 1.54(1.19,1.99) | 0.002 | 1.54(1.18,2.01) | 0.003 |
| High | 1.57(1.21,2.05) | 0.001 | 1.48(1.13,1.94) | 0.01 | 1.74(1.33,2.29) | <0.001 | 1.78(1.37,2.30) | <0.001 |
| P for trend |  | 0.002 |  | 0.01 |  | <0.001 |  | <0.001 |
|  | **BMI** | | | | | | | |
| Under/Normal weight | ref |  | ref |  | ref |  | ref |  |
| Overweight | 0.77(0.58,1.02) | 0.07 | 0.96(0.70,1.31) | 0.78 | 0.88(0.65,1.20) | 0.42 | 0.87(0.64,1.18) | 0.35 |
| Obese | 0.64(0.49,0.83) | 0.001 | 0.68(0.51,0.89) | 0.01 | 0.57(0.44,0.75) | <0.001 | 0.56(0.43,0.73) | <0.001 |
| P for trend |  | 0.002 |  | 0.01 |  | <0.001 |  | <0.001 |
|  | **Blood lipids score classification** | | | | | | | |
| Low | ref |  | ref |  | ref |  | ref |  |
| Moderate | 1.27(1.01,1.62) | 0.04 | 1.11(0.88,1.40) | 0.38 | 1.18(0.93,1.50) | 0.16 | 1.14(0.90,1.44) | 0.26 |
| High | 1.29(1.05,1.59) | 0.02 | 1.01(0.82,1.26) | 0.90 | 1.11(0.88,1.39) | 0.37 | 0.99(0.79,1.25) | 0.95 |
| P for trend |  | 0.02 |  | 0.94 |  | 0.4 |  | 0.9 |
|  | **Blood glucose score classification** | | | | | | | |
| Low | ref |  | ref |  | ref |  | ref |  |
| Moderate | 1.11(0.74,1.67) | 0.61 | 1.15(0.76,1.75) | 0.50 | 1.28(0.85,1.92) | 0.23 | 1.21(0.81,1.82) | 0.35 |
| High | 1.08(0.79,1.49) | 0.60 | 1.00(0.73,1.36) | 1.00 | 1.24(0.90,1.69) | 0.18 | 1.06(0.76,1.46) | 0.73 |
| P for trend |  | 0.8 |  | 0.55 |  | 0.45 |  | 0.77 |
|  | **Blood pressure score classification** | | | | | | | |
| Low | ref |  | ref |  | ref |  | ref |  |
| Moderate | 0.92(0.71,1.20) | 0.54 | 0.96(0.72,1.28) | 0.78 | 1.05(0.77,1.43) | 0.77 | 1.03(0.74,1.42) | 0.86 |
| High | 1.43(1.11,1.83) | 0.01 | 1.14(0.83,1.57) | 0.42 | 1.26(0.91,1.74) | 0.16 | 1.17(0.85,1.62) | 0.33 |
| P for trend |  | <0.001 |  | 0.23 |  | 0.07 |  | 0.2 |

Crude model: Unadjusted model

model 1: Adjusted for age, sex and ethnic

model 2: Additionally adjusted for marital status, educational level, family size, annual family income, smoking, alcohol consumption, and PHQ-9

model 3: Additionally adjusted for vigorous physical activity, BMI, hypertension, hyperlipidemia, Diabetes mellitus

BMI: Body Mass Index; OR: odds ratios; CI: confidence interval;

**Supplementary table 4. Stratified analysis of Chronic Constipation across LE8 score**

| **Character** | **Low** | **Moderate (OR [95% CI])** | **P value** | **High (OR [95% CI])** | **P value** | **P for trend** | **P for interaction** |
| --- | --- | --- | --- | --- | --- | --- | --- |
| **Age group** |  |  |  |  |  |  | 0.66 |
| **20-40** | ref | 0.87(0.37,2.05) | 0.75 | 0.83(0.35,2.00) | 0.68 | 0.65 |  |
| **40-60** | ref | 0.74(0.50,1.09) | 0.12 | 0.89(0.54,1.48) | 0.65 | 0.75 |  |
| **≥60** | ref | 0.60(0.35,1.02) | 0.06 | 0.88(0.46,1.70) | 0.71 | 0.55 |  |
| **Sex** |  |  |  |  |  |  | 0.34 |
| **Female** | ref | 0.72(0.48,1.08) | 0.11 | 0.84(0.52,1.37) | 0.48 | 0.81 |  |
| **Male** | ref | 0.77(0.42,1.42) | 0.4 | 0.62(0.32,1.23) | 0.17 | 0.18 |  |
| **Ethnic** |  |  |  |  |  |  | < 0.001 |
| **White** | ref | 0.56(0.37,0.87) | 0.01 | 0.68(0.42,1.11) | 0.12 | 0.35 |  |
| **Black** | ref | 0.78(0.50,1.23) | 0.28 | 0.86(0.41,1.81) | 0.68 | 0.56 |  |
| **Other** | ref | 3.30(1.67,6.52) | 0.001 | 2.78(1.25,6.15) | 0.01 | 0.22 |  |
| **Marital status** |  |  |  |  |  |  | 0.97 |
| **Married/With partner** | ref | 0.73(0.51,1.04) | 0.08 | 0.82(0.55,1.23) | 0.32 | 0.65 |  |
| **Alone** | ref | 0.77(0.47,1.25) | 0.28 | 0.85(0.43,1.68) | 0.63 | 0.73 |  |
| **Educational level** |  |  |  |  |  |  | 0.58 |
| **≤Highschool** | ref | 0.76(0.51,1.13) | 0.17 | 0.71(0.42,1.21) | 0.2 | 0.2 |  |
| **>Highschool** | ref | 0.71(0.43,1.18) | 0.18 | 0.89(0.54,1.47) | 0.65 | 0.52 |  |
| **Family size** |  |  |  |  |  |  | 0.91 |
| **1-3** | ref | 0.75(0.57,1.00) | 0.05 | 0.83(0.57,1.22) | 0.34 | 0.58 |  |
| **>3** | ref | 0.66(0.36,1.22) | 0.18 | 0.74(0.36,1.52) | 0.39 | 0.59 |  |
| **Annual family income** |  |  |  |  |  |  | 0.62 |
| **Under $20,000** | ref | 0.82(0.44,1.50) | 0.5 | 0.55(0.23,1.27) | 0.16 | 0.17 |  |
| **$20,000 - $35,000** | ref | 0.68(0.43,1.08) | 0.1 | 0.81(0.44,1.50) | 0.49 | 0.52 |  |
| **$35,000 - $75,000** | ref | 0.79(0.45,1.39) | 0.4 | 1.10(0.52,2.33) | 0.8 | 0.51 |  |
| **Over $75,000** | ref | 0.59(0.24,1.45) | 0.24 | 0.67(0.30,1.49) | 0.31 | 0.79 |  |
| **Smoking** |  |  |  |  |  |  | 0.04 |
| **No** | ref | 0.70(0.45,1.10) | 0.12 | 0.87(0.53,1.43) | 0.57 | 0.45 |  |
| **Yes** | ref | 0.73(0.52,1.03) | 0.07 | 0.41(0.18,0.95) | 0.04 | 0.02 |  |
| **Drinking** |  |  |  |  |  |  | 0.13 |
| **No** | ref | 0.55(0.26,1.15) | 0.11 | 0.83(0.32,2.12) | 0.69 | 0.86 |  |
| **Yes** | ref | 0.77(0.55,1.09) | 0.13 | 0.79(0.50,1.24) | 0.29 | 0.4 |  |
| **Weight status** |  |  |  |  |  |  | 0.04 |
| **Under/Normal weight** | ref | 0.54(0.25,1.18) | 0.12 | 0.48(0.19,1.22) | 0.12 | 0.29 |  |
| **Overweight** | ref | 0.69(0.37,1.29) | 0.24 | 0.74(0.31,1.77) | 0.49 | 0.73 |  |
| **Obese** | ref | 0.64(0.43,0.94) | 0.02 | 0.05(0.02,0.19) | <0.0001 | 0.001 |  |
| **PHQ-9 (cut-off by 5)** |  |  |  |  |  |  | 0.02 |
| **No** | ref | 0.72(0.46,1.12) | 0.14 | 0.90(0.56,1.44) | 0.65 | 0.68 |  |
| **Yes** | ref | 0.77(0.51,1.18) | 0.22 | 0.43(0.22,0.86) | 0.02 | 0.02 |  |
| **Vigorous physical activity** |  |  |  |  |  |  | 0.25 |
| **No** | ref | 0.72(0.52,1.00) | 0.05 | 0.66(0.38,1.13) | 0.12 | 0.11 |  |
| **Yes** | ref | 0.93(0.36,2.40) | 0.88 | 1.33(0.53,3.35) | 0.53 | 0.08 |  |

Adjusted for age, sex, ehtnic, marital status, educational level, family size, annual family income, drinking, and PHQ-9

LE8: life’s essential 8; PHQ-9: 9-question Patient Health Questionnaire; BMI: Body Mass Index; OR: odds ratios; CI: confidence interval;

**Supplementary table 5. Stratified analysis of Chronic Diarrhea across LE8 score**

| **Character** | **Low** | **Moderate (OR [95% CI])** | **P value** | **High (OR [95% CI])** | **P value** | **P for trend** | **P for interaction** |
| --- | --- | --- | --- | --- | --- | --- | --- |
| **Age group** |  |  |  |  |  |  | 0.3 |
| **20-40** | ref | 0.88(0.47,1.64) | 0.67 | 0.70(0.34,1.45) | 0.32 | 0.28 |  |
| **40-60** | ref | 0.97(0.71,1.33) | 0.84 | 0.46(0.26,0.82) | 0.01 | 0.003 |  |
| **≥60** | ref | 0.92(0.62,1.37) | 0.67 | 0.37(0.17,0.79) | 0.01 | 0.02 |  |
| **Sex** |  |  |  |  |  |  | 0.13 |
| **Female** | ref | 0.77(0.55,1.08) | 0.12 | 0.39(0.24,0.64) | <0.001 | <0.001 |  |
| **Male** | ref | 1.14(0.76,1.72) | 0.52 | 0.82(0.47,1.42) | 0.47 | 0.35 |  |
| **Ethnic** |  |  |  |  |  |  | 0.71 |
| **White** | ref | 0.94(0.68,1.29) | 0.69 | 0.54(0.32,0.94) | 0.03 | 0.01 |  |
| **Black** | ref | 0.81(0.49,1.31) | 0.37 | 0.74(0.29,1.91) | 0.52 | 0.45 |  |
| **Other** | ref | 0.99(0.59,1.68) | 0.97 | 0.48(0.26,0.90) | 0.02 | 0.005 |  |
| **Marital status** |  |  |  |  |  |  | 0.76 |
| **Married/With partner** | ref | 0.88(0.65,1.18) | 0.37 | 0.48(0.30,0.77) | 0.003 | <0.001 |  |
| **Alone** | ref | 1.09(0.71,1.65) | 0.69 | 0.79(0.41,1.49) | 0.45 | 0.41 |  |
| **Educational level** |  |  |  |  |  |  | 0.16 |
| **≤Highschool** | ref | 0.82(0.62,1.07) | 0.14 | 0.56(0.35,0.88) | 0.01 | 0.01 |  |
| **>Highschool** | ref | 1.23(0.76,2.00) | 0.39 | 0.67(0.36,1.24) | 0.2 | 0.01 |  |
| **Family size** |  |  |  |  |  |  | 0.88 |
| **1-3** | ref | 0.93(0.69,1.25) | 0.61 | 0.54(0.33,0.89) | 0.02 | 0.01 |  |
| **>3** | ref | 0.90(0.58,1.39) | 0.62 | 0.55(0.32,0.94) | 0.03 | 0.01 |  |
| **Annual family income** |  |  |  |  |  |  | 0.32 |
| **Under $20,000** | ref | 0.93(0.66,1.31) | 0.67 | 1.10(0.56,2.13) | 0.78 | 0.85 |  |
| **$20,000 - $35,000** | ref | 1.03(0.64,1.67) | 0.89 | 0.60(0.29,1.24) | 0.16 | 0.15 |  |
| **$35,000 - $75,000** | ref | 0.80(0.53,1.20) | 0.27 | 0.39(0.21,0.71) | 0.003 | 0.002 |  |
| **Over $75,000** | ref | 1.17(0.60,2.27) | 0.64 | 0.57(0.29,1.11) | 0.09 | 0.002 |  |
| **Smoking** |  |  |  |  |  |  | 0.02 |
| **No** | ref | 0.78(0.50,1.19) | 0.24 | 0.36(0.21,0.64) | <0.001 | <0.0001 |  |
| **Yes** | ref | 0.94(0.69,1.28) | 0.7 | 0.96(0.49,1.89) | 0.9 | 0.83 |  |
| **Drinking** |  |  |  |  |  |  | 0.26 |
| **No** | ref | 0.94(0.48,1.83) | 0.85 | 0.37(0.14,0.98) | 0.05 | 0.03 |  |
| **Yes** | ref | 0.93(0.72,1.21) | 0.59 | 0.58(0.37,0.90) | 0.02 | 0.01 |  |
| **Weight status** |  |  |  |  |  |  | 0.8 |
| **Under/Normal weight** | ref | 0.87(0.25,3.00) | 0.83 | 0.65(0.17,2.44) | 0.51 | 0.27 |  |
| **Overweight** | ref | 0.95(0.50,1.79) | 0.87 | 0.51(0.26,1.01) | 0.05 | 0.01 |  |
| **Obese** | ref | 1.12(0.86,1.47) | 0.37 | 0.54(0.21,1.39) | 0.19 | 0.94 |  |
| **PHQ-9 (cut-off by 5)** |  |  |  |  |  |  | 0.54 |
| **No** | ref | 0.89(0.64,1.25) | 0.5 | 0.55(0.35,0.87) | 0.01 | 0.003 |  |
| **Yes** | ref | 1.04(0.71,1.55) | 0.82 | 0.44(0.17,1.14) | 0.09 | 0.13 |  |
| **Vigorous physical activity** |  |  |  |  |  |  | 0.25 |
| **No** | ref | 1.04(0.79,1.37) | 0.78 | 0.52(0.31,0.89) | 0.02 | 0.03 |  |
| **Yes** | ref | 0.65(0.35,1.20) | 0.16 | 0.42(0.22,0.80) | 0.01 | 0.004 |  |

Adjusted for age, sex, ehtnic, marital status, educational level, family size, annual family income, drinking, and PHQ-9

LE8: life’s essential 8; PHQ-9: 9-question Patient Health Questionnaire; BMI: Body Mass Index; OR: odds ratios; CI: confidence interval;

**Supplementary table 6. Stratified analysis of Fecal Incontinence across LE8 score**

| **Character** | **Low** | **Moderate (OR [95% CI])** | **P value** | **High (OR [95% CI])** | **P value** | **P for trend** | **P for interaction** |
| --- | --- | --- | --- | --- | --- | --- | --- |
| **Age group** |  |  |  |  |  |  | 0.11 |
| **20-40** | ref | 0.51(0.24,1.11) | 0.09 | 0.39(0.18,0.87) | 0.02 | 0.05 |  |
| **40-60** | ref | 0.90(0.57,1.42) | 0.64 | 0.64(0.35,1.17) | 0.14 | 0.13 |  |
| **≥60** | ref | 1.23(0.83,1.82) | 0.28 | 1.18(0.67,2.07) | 0.55 | 0.54 |  |
| **Sex** |  |  |  |  |  |  | 0.79 |
| **Female** | ref | 0.84(0.64,1.11) | 0.22 | 0.68(0.43,1.09) | 0.11 | 0.11 |  |
| **Male** | ref | 1.09(0.71,1.66) | 0.7 | 0.84(0.43,1.65) | 0.61 | 0.57 |  |
| **Ethnic** |  |  |  |  |  |  | 0.6 |
| **White** | ref | 1.02(0.76,1.38) | 0.87 | 0.81(0.56,1.19) | 0.28 | 0.21 |  |
| **Black** | ref | 0.88(0.48,1.62) | 0.66 | 0.36(0.10,1.29) | 0.11 | 0.23 |  |
| **Other** | ref | 0.67(0.33,1.35) | 0.25 | 0.60(0.25,1.46) | 0.25 | 0.3 |  |
| **Marital status** |  |  |  |  |  |  | 0.03 |
| **Married/With partner** | ref | 1.07(0.77,1.49) | 0.68 | 1.05(0.69,1.60) | 0.81 | 0.87 |  |
| **Alone** | ref | 0.87(0.57,1.31) | 0.48 | 0.42(0.22,0.78) | 0.01 | 0.01 |  |
| **Educational level** |  |  |  |  |  |  | 0.39 |
| **≤Highschool** | ref | 1.16(0.79,1.69) | 0.44 | 0.84(0.36,1.96) | 0.68 | 0.95 |  |
| **>Highschool** | ref | 0.74(0.51,1.08) | 0.12 | 0.63(0.39,1.02) | 0.06 | 0.1 |  |
| **Family size** |  |  |  |  |  |  | 0.69 |
| **1-3** | ref | 0.90(0.66,1.23) | 0.5 | 0.71(0.48,1.07) | 0.1 | 0.08 |  |
| **>3** | ref | 1.10(0.60,2.01) | 0.75 | 0.90(0.34,2.37) | 0.83 | 0.75 |  |
| **Annual family income** |  |  |  |  |  |  | 0.3 |
| **Under $20,000** | ref | 1.35(0.78,2.32) | 0.27 | 0.66(0.26,1.69) | 0.38 | 0.86 |  |
| **$20,000 - $35,000** | ref | 0.98(0.56,1.71) | 0.95 | 0.52(0.25,1.09) | 0.08 | 0.15 |  |
| **$35,000 - $75,000** | ref | 0.66(0.42,1.03) | 0.06 | 0.57(0.31,1.04) | 0.07 | 0.1 |  |
| **Over $75,000** | ref | 0.99(0.49, 1.99) | 0.98 | 0.92(0.37, 2.26) | 0.85 | 0.8 |  |
| **Smoking** |  |  |  |  |  |  | 0.33 |
| **No** | ref | 1.32(0.77,2.25) | 0.3 | 1.02(0.55,1.90) | 0.94 | 0.43 |  |
| **Yes** | ref | 0.86(0.63,1.18) | 0.34 | 0.70(0.41,1.19) | 0.18 | 0.17 |  |
| **Drinking** |  |  |  |  |  |  | 0.07 |
| **No** | ref | 1.82(0.69,4.83) | 0.22 | 1.62(0.53,4.98) | 0.39 | 0.63 |  |
| **Yes** | ref | 0.89(0.67,1.16) | 0.37 | 0.67(0.46,0.96) | 0.03 | 0.02 |  |
| **Weight status** |  |  |  |  |  |  | 0.52 |
| **Under/Normal weight** | ref | 1.01(0.45, 2.28) | 0.98 | 0.68(0.28, 1.68) | 0.39 | 0.13 |  |
| **Overweight** | ref | 0.72(0.33,1.59) | 0.41 | 0.75(0.24,2.35) | 0.61 | 0.73 |  |
| **Obese** | ref | 1.11(0.76,1.61) | 0.59 | 0.57(0.14,2.41) | 0.44 | 0.95 |  |
| **PHQ-9 (cut-off by 5)** |  |  |  |  |  |  | 0.04 |
| **No** | ref | 1.17(0.78,1.73) | 0.44 | 1.10(0.67,1.80) | 0.7 | 0.91 |  |
| **Yes** | ref | 0.82(0.58,1.17) | 0.27 | 0.33(0.13,0.85) | 0.02 | 0.01 |  |
| **Vigorous physical activity** |  |  |  |  |  |  | 0.11 |
| **No** | ref | 0.90(0.68,1.19) | 0.44 | 0.93(0.64,1.34) | 0.67 | 0.61 |  |
| **Yes** | ref | 1.22(0.53, 2.85) | 0.63 | 0.80(0.33, 1.97) | 0.62 | 0.17 |  |

Adjusted for age, sex, ehtnic, marital status, educational level, family size, annual family income, drinking, and PHQ-9

LE8: life’s essential 8; PHQ-9: 9-question Patient Health Questionnaire; BMI: Body Mass Index; OR: odds ratios; CI: confidence interval;

**Supplementary table 7. Stratified analysis of Chronic Constipation across Health Behaviors score**

| **Character** | **Low** | **Moderate (OR [95% CI])** | **P value** | **High (OR [95% CI])** | **P value** | **P for trend** | **P for interaction** |
| --- | --- | --- | --- | --- | --- | --- | --- |
| **Age group** |  |  |  |  |  |  | 0.19 |
| **20-40** | ref | 0.91(0.63,1.32) | 0.61 | 0.57(0.35,0.93) | 0.03 | 0.03 |  |
| **40-60** | ref | 1.05(0.73,1.51) | 0.78 | 1.09(0.65,1.82) | 0.74 | 0.74 |  |
| **≥60** | ref | 0.54(0.36,0.80) | 0.003 | 0.52(0.31,0.86) | 0.01 | 0.03 |  |
| **Sex** |  |  |  |  |  |  | 0.24 |
| **Female** | ref | 0.78(0.58,1.04) | 0.09 | 0.70(0.48,1.01) | 0.05 | 0.06 |  |
| **Male** | ref | 1.16(0.80,1.69) | 0.43 | 0.71(0.37,1.35) | 0.29 | 0.32 |  |
| **Ethnic** |  |  |  |  |  |  | 0.35 |
| **White** | ref | 0.75(0.56,1.00) | 0.05 | 0.62(0.42,0.91) | 0.02 | 0.02 |  |
| **Black** | ref | 0.83(0.59,1.16) | 0.26 | 0.87(0.52,1.45) | 0.58 | 0.42 |  |
| **Other** | ref | 1.52(0.90,2.56) | 0.11 | 1.16(0.55,2.45) | 0.68 | 0.8 |  |
| **Marital status** |  |  |  |  |  |  | 0.7 |
| **Married/With partner** | ref | 0.89(0.68,1.17) | 0.39 | 0.69(0.46,1.03) | 0.07 | 0.06 |  |
| **Alone** | ref | 0.84(0.60,1.17) | 0.28 | 0.80(0.51,1.26) | 0.33 | 0.31 |  |
| **Educational level** |  |  |  |  |  |  | 0.94 |
| **≤Highschool** | ref | 0.91(0.65,1.25) | 0.54 | 0.83(0.52,1.34) | 0.44 | 0.43 |  |
| **>Highschool** | ref | 0.86(0.62,1.21) | 0.38 | 0.68(0.43,1.08) | 0.1 | 0.08 |  |
| **Family size** |  |  |  |  |  |  | 0.47 |
| **1-3** | ref | 0.91(0.70,1.18) | 0.45 | 0.80(0.55,1.16) | 0.23 | 0.23 |  |
| **>3** | ref | 0.77(0.52,1.16) | 0.21 | 0.55(0.32,0.94) | 0.03 | 0.03 |  |
| **Annual family income** |  |  |  |  |  |  | 1 |
| **Under $20,000** | ref | 0.88(0.60,1.28) | 0.49 | 0.76(0.47,1.23) | 0.26 | 0.24 |  |
| **$20,000 - $35,000** | ref | 0.93(0.61,1.42) | 0.72 | 0.78(0.44,1.38) | 0.39 | 0.4 |  |
| **$35,000 - $75,000** | ref | 0.88(0.60,1.29) | 0.51 | 0.72(0.41,1.27) | 0.25 | 0.24 |  |
| **Over $75,000** | ref | 0.77(0.48,1.22) | 0.25 | 0.64(0.38,1.08) | 0.09 | 0.12 |  |
| **Smoking** |  |  |  |  |  |  | 0.49 |
| **No** | ref | 0.65(0.45,0.93) | 0.02 | 0.53(0.35,0.80) | 0.003 | 0.01 |  |
| **Yes** | ref | 0.82(0.59,1.15) | 0.25 | 0.52(0.27,1.02) | 0.06 | 0.08 |  |
| **Drinking** |  |  |  |  |  |  | 0.01 |
| **No** | ref | 0.30(0.17,0.54) | <0.001 | 0.35(0.17,0.69) | 0.004 | 0.04 |  |
| **Yes** | ref | 0.96(0.76,1.21) | 0.72 | 0.75(0.54,1.03) | 0.08 | 0.08 |  |
| **Weight status** |  |  |  |  |  |  | 0.31 |
| **Under/Normal weight** | ref | 0.85(0.56,1.28) | 0.42 | 0.63(0.36,1.10) | 0.1 | 0.09 |  |
| **Overweight** | ref | 0.93(0.66,1.31) | 0.66 | 1.00(0.61,1.63) | 0.99 | 0.97 |  |
| **Obese** | ref | 0.86(0.55,1.34) | 0.48 | 0.52(0.28,0.97) | 0.04 | 0.05 |  |
| **PHQ-9 (cut-off by 5)** |  |  |  |  |  |  | 0.66 |
| **No** | ref | 0.85(0.63,1.16) | 0.3 | 0.74(0.53,1.04) | 0.08 | 0.08 |  |
| **Yes** | ref | 0.88(0.61,1.29) | 0.51 | 0.59(0.31,1.14) | 0.11 | 0.12 |  |
| **Vigorous physical activity** |  |  |  |  |  |  | 0.04 |
| **No** | ref | 0.78(0.61,0.98) | 0.04 | 0.67(0.43,1.03) | 0.07 | 0.05 |  |
| **Yes** | ref | 1.50(0.94,2.40) | 0.09 | 1.13(0.61,2.08) | 0.7 | 0.76 |  |

Adjusted for age, sex, ehtnic, marital status, educational level, family size, annual family income, drinking, and PHQ-9

LE8: life’s essential 8; PHQ-9: 9-question Patient Health Questionnaire; BMI: Body Mass Index; OR: odds ratios; CI: confidence interval;

**Supplementary table 8. Stratified analysis of Chronic Diarrhea across Health Behaviors score**

| **Character** | **Low** | **Moderate (OR [95% CI])** | **P value** | **High (OR [95% CI])** | **P value** | **P for trend** | **P for interaction** |
| --- | --- | --- | --- | --- | --- | --- | --- |
| **Age group** |  |  |  |  |  |  | 0.61 |
| **20-40** | ref | 0.92(0.63,1.36) | 0.68 | 0.99(0.53,1.83) | 0.97 | 0.97 |  |
| **40-60** | ref | 1.19(0.80,1.75) | 0.38 | 0.84(0.48,1.48) | 0.54 | 0.47 |  |
| **≥60** | ref | 1.10(0.70,1.72) | 0.67 | 0.93(0.55,1.56) | 0.77 | 0.65 |  |
| **Sex** |  |  |  |  |  |  | 0.55 |
| **Female** | ref | 1.09(0.78,1.54) | 0.59 | 0.86(0.57,1.31) | 0.47 | 0.42 |  |
| **Male** | ref | 1.08(0.73,1.59) | 0.7 | 0.98(0.62,1.56) | 0.94 | 0.91 |  |
| **Ethnic** |  |  |  |  |  |  | 0.81 |
| **White** | ref | 1.10(0.77,1.57) | 0.6 | 0.92(0.59,1.44) | 0.71 | 0.61 |  |
| **Black** | ref | 0.92(0.57,1.47) | 0.71 | 0.81(0.39,1.70) | 0.57 | 0.58 |  |
| **Other** | ref | 1.09(0.69,1.73) | 0.71 | 0.85(0.49,1.49) | 0.56 | 0.48 |  |
| **Marital status** |  |  |  |  |  |  | 0.9 |
| **Married/With partner** | ref | 1.11(0.76,1.61) | 0.57 | 0.91(0.51,1.61) | 0.73 | 0.64 |  |
| **Alone** | ref | 1.12(0.77,1.62) | 0.56 | 1.02(0.66,1.58) | 0.92 | 0.88 |  |
| **Educational level** |  |  |  |  |  |  | 0.91 |
| **≤Highschool** | ref | 1.15(0.82,1.61) | 0.4 | 1.00(0.64,1.57) | 0.99 | 0.87 |  |
| **>Highschool** | ref | 1.01(0.66,1.53) | 0.97 | 0.83(0.53,1.31) | 0.42 | 0.29 |  |
| **Family size** |  |  |  |  |  |  | 0.87 |
| **1-3** | ref | 1.03(0.74,1.43) | 0.86 | 0.87(0.57,1.32) | 0.5 | 0.45 |  |
| **>3** | ref | 1.19(0.77,1.83) | 0.42 | 0.99(0.55,1.78) | 0.97 | 1 |  |
| **Annual family income** |  |  |  |  |  |  | 0.76 |
| **Under $20,000** | ref | 1.36(0.94,1.96) | 0.1 | 1.29(0.74,2.27) | 0.36 | 0.22 |  |
| **$20,000 - $35,000** | ref | 1.04(0.69,1.57) | 0.84 | 1.04(0.68,1.59) | 0.87 | 0.86 |  |
| **$35,000 - $75,000** | ref | 1.20(0.72,2.01) | 0.48 | 0.90(0.49,1.66) | 0.73 | 0.65 |  |
| **Over $75,000** | ref | 0.79(0.41,1.50) | 0.46 | 0.64(0.32,1.29) | 0.2 | 0.19 |  |
| **Smoking** |  |  |  |  |  |  | 0.32 |
| **No** | ref | 1.05(0.65,1.70) | 0.85 | 0.84(0.50,1.40) | 0.49 | 0.13 |  |
| **Yes** | ref | 1.07(0.75,1.53) | 0.71 | 1.10(0.60,2.00) | 0.75 | 0.72 |  |
| **Drinking** |  |  |  |  |  |  | 0.54 |
| **No** | ref | 1.94(0.99,3.81) | 0.05 | 1.78(0.70,4.54) | 0.22 | 0.36 |  |
| **Yes** | ref | 1.04(0.78,1.39) | 0.76 | 0.88(0.62,1.25) | 0.46 | 0.44 |  |
| **Weight status** |  |  |  |  |  |  | 0.85 |
| **Under/Normal weight** | ref | 1.15(0.67,1.98) | 0.6 | 1.07(0.48,2.35) | 0.87 | 0.89 |  |
| **Overweight** | ref | 0.88(0.58,1.33) | 0.53 | 0.65(0.39,1.09) | 0.1 | 0.09 |  |
| **Obese** | ref | 1.14(0.76,1.72) | 0.51 | 1.10(0.67,1.80) | 0.69 | 0.69 |  |
| **PHQ-9 (cut-off by 5)** |  |  |  |  |  |  | 0.76 |
| **No** | ref | 1.04(0.71,1.51) | 0.84 | 0.83(0.52,1.33) | 0.43 | 0.33 |  |
| **Yes** | ref | 1.21(0.80,1.83) | 0.34 | 1.30(0.79,2.16) | 0.3 | 0.22 |  |
| **Vigorous physical activity** |  |  |  |  |  |  | 0.38 |
| **No** | ref | 1.22(0.87,1.71) | 0.24 | 0.96(0.63,1.46) | 0.85 | 0.99 |  |
| **Yes** | ref | 0.83(0.55,1.25) | 0.35 | 0.75(0.47,1.20) | 0.22 | 0.27 |  |

Adjusted for age, sex, ehtnic, marital status, educational level, family size, annual family income, drinking, and PHQ-9

LE8: life’s essential 8; PHQ-9: 9-question Patient Health Questionnaire; BMI: Body Mass Index; OR: odds ratios; CI: confidence interval;

**Supplementary table 9. Stratified analysis of Fecal Incontinence across Health Behaviors score**

| **Character** | **Low** | **Moderate (OR [95% CI])** | **P value** | **High (OR [95% CI])** | **P value** | **P for trend** | **P for interaction** |
| --- | --- | --- | --- | --- | --- | --- | --- |
| **Age group** |  |  |  |  |  |  | 0.17 |
| **20-40** | ref | 0.65(0.40,1.07) | 0.09 | 0.41(0.18,0.94) | 0.04 | 0.03 |  |
| **40-60** | ref | 1.10(0.70,1.71) | 0.68 | 0.78(0.41,1.46) | 0.42 | 0.39 |  |
| **≥60** | ref | 0.83(0.55,1.25) | 0.36 | 0.82(0.56,1.18) | 0.27 | 0.33 |  |
| **Sex** |  |  |  |  |  |  | 0.77 |
| **Female** | ref | 0.79(0.58,1.08) | 0.13 | 0.64(0.41,1.00) | 0.05 | 0.05 |  |
| **Male** | ref | 1.05(0.66,1.67) | 0.83 | 0.85(0.47,1.55) | 0.59 | 0.57 |  |
| **Ethnic** |  |  |  |  |  |  | 0.63 |
| **White** | ref | 0.90(0.63,1.27) | 0.53 | 0.75(0.47,1.21) | 0.23 | 0.22 |  |
| **Black** | ref | 1.07(0.66,1.74) | 0.77 | 0.49(0.21,1.14) | 0.1 | 0.22 |  |
| **Other** | ref | 0.75(0.44,1.27) | 0.27 | 0.63(0.36,1.11) | 0.1 | 0.11 |  |
| **Marital status** |  |  |  |  |  |  | 0.08 |
| **Married/With partner** | ref | 1.14(0.73,1.79) | 0.55 | 0.94(0.56,1.59) | 0.81 | 0.68 |  |
| **Alone** | ref | 0.66(0.47,0.92) | 0.02 | 0.53(0.33,0.83) | 0.01 | 0.01 |  |
| **Educational level** |  |  |  |  |  |  | 0.09 |
| **≤Highschool** | ref | 0.79(0.56,1.13) | 0.19 | 0.92(0.53,1.59) | 0.76 | 0.63 |  |
| **>Highschool** | ref | 1.02(0.69, 1.49) | 0.93 | 0.68(0.43, 1.09) | 0.11 | 0.04 |  |
| **Family size** |  |  |  |  |  |  | 0.86 |
| **1-3** | ref | 0.85(0.65,1.12) | 0.24 | 0.70(0.48,1.04) | 0.08 | 0.07 |  |
| **>3** | ref | 0.94(0.56,1.60) | 0.82 | 0.74(0.37,1.48) | 0.38 | 0.37 |  |
| **Annual family income** |  |  |  |  |  |  | 0.88 |
| **Under $20,000** | ref | 0.87(0.58,1.33) | 0.52 | 0.90(0.43,1.89) | 0.78 | 0.69 |  |
| **$20,000 - $35,000** | ref | 0.80(0.48,1.31) | 0.36 | 0.81(0.47,1.39) | 0.43 | 0.42 |  |
| **$35,000 - $75,000** | ref | 0.87(0.55,1.36) | 0.53 | 0.57(0.33,0.98) | 0.04 | 0.03 |  |
| **Over $75,000** | ref | 1.15(0.52, 2.56) | 0.73 | 0.86(0.34, 2.20) | 0.75 | 0.52 |  |
| **Smoking** |  |  |  |  |  |  | 0.82 |
| **No** | ref | 0.94(0.53,1.65) | 0.82 | 0.71(0.40,1.26) | 0.24 | 0.08 |  |
| **Yes** | ref | 0.86(0.58,1.26) | 0.42 | 0.80(0.44,1.43) | 0.43 | 0.4 |  |
| **Drinking** |  |  |  |  |  |  | 0.41 |
| **No** | ref | 0.49(0.23,1.02) | 0.06 | 0.54(0.23,1.28) | 0.16 | 0.33 |  |
| **Yes** | ref | 0.94(0.72,1.23) | 0.64 | 0.73(0.50,1.06) | 0.09 | 0.08 |  |
| **Weight status** |  |  |  |  |  |  | 0.95 |
| **Under/Normal weight** | ref | 0.90(0.48, 1.66) | 0.72 | 0.65(0.34, 1.25) | 0.19 | 0.16 |  |
| **Overweight** | ref | 0.78(0.46,1.32) | 0.34 | 0.70(0.35,1.42) | 0.31 | 0.34 |  |
| **Obese** | ref | 0.98(0.72,1.33) | 0.9 | 0.78(0.48,1.25) | 0.29 | 0.28 |  |
| **PHQ-9 (cut-off by 5)** |  |  |  |  |  |  | 0.5 |
| **No** | ref | 1.03(0.68,1.55) | 0.9 | 0.80(0.50,1.26) | 0.32 | 0.19 |  |
| **Yes** | ref | 0.80(0.55,1.16) | 0.23 | 0.71(0.40,1.27) | 0.24 | 0.19 |  |
| **Vigorous physical activity** |  |  |  |  |  |  | 0.11 |
| **No** | ref | 0.82(0.63,1.06) | 0.13 | 0.81(0.59,1.10) | 0.17 | 0.16 |  |
| **Yes** | ref | 1.29(0.68, 2.43) | 0.42 | 0.83(0.38, 1.81) | 0.64 | 0.3 |  |

Adjusted for age, sex, ehtnic, marital status, educational level, family size, annual family income, drinking, and PHQ-9

LE8: life’s essential 8; PHQ-9: 9-question Patient Health Questionnaire; BMI: Body Mass Index; OR: odds ratios; CI: confidence interval;

**Supplementary table 10. Stratified analysis of Chronic Constipation across Health Factors score**

| **Character** | **Low** | **Moderate (OR [95% CI])** | **P value** | **High (OR [95% CI])** | **P value** | **P for trend** | **P for interaction** |
| --- | --- | --- | --- | --- | --- | --- | --- |
| **Age group** |  |  |  |  |  |  | 0.3 |
| **20-40** | ref | 0.86(0.40,1.82) | 0.68 | 1.30(0.61,2.75) | 0.49 | 0.05 |  |
| **40-60** | ref | 1.07(0.60,1.92) | 0.8 | 1.20(0.76,1.88) | 0.42 | 0.38 |  |
| **≥60** | ref | 0.93(0.63,1.37) | 0.7 | 1.88(1.02,3.48) | 0.04 | 0.09 |  |
| **Sex** |  |  |  |  |  |  | 0.34 |
| **Female** | ref | 1.11(0.76,1.61) | 0.58 | 1.48(1.02,2.14) | 0.04 | 0.03 |  |
| **Male** | ref | 0.71(0.38,1.33) | 0.27 | 1.10(0.58,2.08) | 0.76 | 0.37 |  |
| **Ethnic** |  |  |  |  |  |  | 0.92 |
| **White** | ref | 0.95(0.60,1.51) | 0.84 | 1.38(0.90,2.12) | 0.14 | 0.04 |  |
| **Black** | ref | 0.98(0.60,1.61) | 0.94 | 1.30(0.74,2.27) | 0.35 | 0.28 |  |
| **Other** | ref | 1.18(0.55,2.49) | 0.66 | 1.67(0.83,3.36) | 0.14 | 0.05 |  |
| **Marital status** |  |  |  |  |  |  | 0.64 |
| **Married/With partner** | ref | 1.10(0.69,1.75) | 0.67 | 1.43(0.88,2.32) | 0.14 | 0.06 |  |
| **Alone** | ref | 0.86(0.51,1.45) | 0.56 | 1.34(0.78,2.31) | 0.28 | 0.11 |  |
| **Educational level** |  |  |  |  |  |  | 0.96 |
| **≤Highschool** | ref | 0.90(0.59,1.37) | 0.62 | 1.19(0.74,1.92) | 0.46 | 0.31 |  |
| **>Highschool** | ref | 1.09(0.67,1.79) | 0.72 | 1.60(0.97,2.66) | 0.07 | 0.03 |  |
| **Family size** |  |  |  |  |  |  | 0.2 |
| **1-3** | ref | 1.11(0.78,1.56) | 0.55 | 1.37(0.99,1.88) | 0.06 | 0.05 |  |
| **>3** | ref | 0.71(0.38,1.30) | 0.26 | 1.27(0.64,2.54) | 0.49 | 0.08 |  |
| **Annual family income** |  |  |  |  |  |  | 0.54 |
| **Under $20,000** | ref | 1.40(0.76,2.56) | 0.27 | 1.43(0.71,2.87) | 0.3 | 0.4 |  |
| **$20,000 - $35,000** | ref | 0.74(0.42,1.32) | 0.3 | 1.09(0.58,2.05) | 0.79 | 0.47 |  |
| **$35,000 - $75,000** | ref | 0.89(0.52,1.53) | 0.67 | 1.32(0.80,2.17) | 0.27 | 0.11 |  |
| **Over $75,000** | ref | 1.25(0.58,2.69) | 0.56 | 1.92(0.86,4.26) | 0.11 | 0.03 |  |
| **Smoking** |  |  |  |  |  |  | 0.004 |
| **No** | ref | 1.55(1.02,2.37) | 0.04 | 2.55(1.75,3.72) | <0.0001 | <0.0001 |  |
| **Yes** | ref | 0.63(0.39,1.02) | 0.06 | 0.75(0.43,1.31) | 0.3 | 0.62 |  |
| **Drinking** |  |  |  |  |  |  | 0.02 |
| **No** | ref | 0.90(0.41,2.00) | 0.79 | 2.28(1.05,4.94) | 0.04 | 0.01 |  |
| **Yes** | ref | 1.01(0.72,1.41) | 0.96 | 1.31(0.88,1.94) | 0.18 | 0.11 |  |
| **Weight status** |  |  |  |  |  |  | 0.15 |
| **Under/Normal weight** | ref | 0.37(0.09,1.58) | 0.17 | 0.38(0.08,1.82) | 0.22 | 0.83 |  |
| **Overweight** | ref | 0.76(0.31,1.86) | 0.54 | 1.07(0.39,2.89) | 0.9 | 0.35 |  |
| **Obese** | ref | 0.85(0.61,1.18) | 0.33 | 0.64(0.34,1.23) | 0.17 | 0.14 |  |
| **PHQ-9 (cut-off by 5)** |  |  |  |  |  |  | 0.18 |
| **No** | ref | 0.96(0.65,1.44) | 0.85 | 1.50(0.99,2.28) | 0.05 | 0.01 |  |
| **Yes** | ref | 1.08(0.61,1.90) | 0.79 | 1.15(0.67,1.98) | 0.59 | 0.58 |  |
| **Vigorous physical activity** |  |  |  |  |  |  | 0.89 |
| **No** | ref | 0.93(0.66,1.33) | 0.7 | 1.33(0.92,1.93) | 0.12 | 0.06 |  |
| **Yes** | ref | 1.10(0.51,2.39) | 0.8 | 1.53(0.71,3.28) | 0.26 | 0.09 |  |

Adjusted for age, sex, ehtnic, marital status, educational level, family size, annual family income, drinking, and PHQ-9

LE8: life’s essential 8; PHQ-9: 9-question Patient Health Questionnaire; BMI: Body Mass Index; OR: odds ratios; CI: confidence interval;

**Supplementary table 11. Stratified analysis of Chronic Diarrhea across Health Factors score**

| **Character** | **Low** | **Moderate (OR [95% CI])** | **P value** | **High (OR [95% CI])** | **P value** | **P for trend** | **P for interaction** |
| --- | --- | --- | --- | --- | --- | --- | --- |
| **Age group** |  |  |  |  |  |  | 0.63 |
| **20-40** | ref | 0.62(0.33,1.20) | 0.15 | 0.51(0.27,0.97) | 0.04 | 0.06 |  |
| **40-60** | ref | 0.87(0.60,1.25) | 0.43 | 0.60(0.37,0.97) | 0.04 | 0.03 |  |
| **≥60** | ref | 1.10(0.70,1.71) | 0.67 | 0.65(0.36,1.19) | 0.16 | 0.3 |  |
| **Sex** |  |  |  |  |  |  | 0.03 |
| **Female** | ref | 0.62(0.40,0.95) | 0.03 | 0.48(0.31,0.74) | 0.002 | 0.002 |  |
| **Male** | ref | 1.32(0.90,1.94) | 0.15 | 0.93(0.56,1.52) | 0.76 | 0.42 |  |
| **Ethnic** |  |  |  |  |  |  | 0.61 |
| **White** | ref | 0.86(0.60,1.23) | 0.4 | 0.62(0.41,0.93) | 0.02 | 0.01 |  |
| **Black** | ref | 0.73(0.46,1.17) | 0.18 | 0.64(0.41,1.01) | 0.06 | 0.07 |  |
| **Other** | ref | 1.17(0.66,2.05) | 0.58 | 0.74(0.37,1.45) | 0.37 | 0.13 |  |
| **Marital status** |  |  |  |  |  |  | 0.78 |
| **Married/With partner** | ref | 0.83(0.63,1.09) | 0.17 | 0.57(0.38,0.84) | 0.01 | 0.004 |  |
| **Alone** | ref | 1.06(0.66,1.71) | 0.8 | 0.86(0.51,1.44) | 0.55 | 0.42 |  |
| **Educational level** |  |  |  |  |  |  | 0.12 |
| **≤Highschool** | ref | 1.11(0.86,1.43) | 0.4 | 0.84(0.56,1.25) | 0.37 | 0.29 |  |
| **>Highschool** | ref | 0.70(0.46,1.06) | 0.09 | 0.49(0.33,0.73) | <0.001 | <0.001 |  |
| **Family size** |  |  |  |  |  |  | 0.71 |
| **1-3** | ref | 0.86(0.63,1.19) | 0.36 | 0.67(0.46,0.99) | 0.04 | 0.03 |  |
| **>3** | ref | 0.94(0.60,1.47) | 0.78 | 0.59(0.35,0.98) | 0.04 | 0.02 |  |
| **Annual family income** |  |  |  |  |  |  | 0.04 |
| **Under $20,000** | ref | 0.92(0.63,1.35) | 0.66 | 0.81(0.49,1.34) | 0.4 | 0.39 |  |
| **$20,000 - $35,000** | ref | 1.94(1.22,3.09) | 0.01 | 1.18(0.72,1.93) | 0.51 | 0.76 |  |
| **$35,000 - $75,000** | ref | 0.57(0.36,0.90) | 0.02 | 0.42(0.22,0.80) | 0.01 | 0.01 |  |
| **Over $75,000** | ref | 0.95(0.53,1.69) | 0.86 | 0.66(0.33,1.33) | 0.24 | 0.13 |  |
| **Smoking** |  |  |  |  |  |  | 0.16 |
| **No** | ref | 0.72(0.46,1.13) | 0.15 | 0.46(0.29,0.71) | 0.001 | <0.001 |  |
| **Yes** | ref | 1.11(0.82,1.51) | 0.48 | 0.89(0.54,1.49) | 0.66 | 0.49 |  |
| **Drinking** |  |  |  |  |  |  | 0.5 |
| **No** | ref | 0.71(0.39,1.28) | 0.25 | 0.54(0.30,0.96) | 0.04 | 0.04 |  |
| **Yes** | ref | 0.92(0.71,1.18) | 0.49 | 0.66(0.48,0.89) | 0.01 | 0.005 |  |
| **Weight status** |  |  |  |  |  |  | 0.09 |
| **Under/Normal weight** | ref | 706286.91(386360.12,1291130.15) | <0.0001 | 488323.03(262052.98, 909966.29) | <0.0001 | 0.16 |  |
| **Overweight** | ref | 2.00(0.83,4.82) | 0.12 | 1.59(0.73,3.49) | 0.23 | 0.88 |  |
| **Obese** | ref | 0.94(0.70,1.26) | 0.68 | 0.92(0.37,2.31) | 0.86 | 0.71 |  |
| **PHQ-9 (cut-off by 5)** |  |  |  |  |  |  | 0.15 |
| **No** | ref | 1.03(0.76,1.41) | 0.84 | 0.74(0.53,1.03) | 0.07 | 0.02 |  |
| **Yes** | ref | 0.62(0.40,0.95) | 0.03 | 0.46(0.26,0.81) | 0.01 | 0.01 |  |
| **Vigorous physical activity** |  |  |  |  |  |  | 0.76 |
| **No** | ref | 0.92(0.69,1.23) | 0.57 | 0.62(0.45,0.84) | 0.003 | 0.002 |  |
| **Yes** | ref | 0.81(0.49,1.35) | 0.41 | 0.63(0.35,1.16) | 0.14 | 0.11 |  |

Adjusted for age, sex, ehtnic, marital status, educational level, family size, annual family income, drinking, and PHQ-9

LE8: life’s essential 8; PHQ-9: 9-question Patient Health Questionnaire; BMI: Body Mass Index; OR: odds ratios; CI: confidence interval;

**Supplementary table 12. Stratified analysis of Fecal Incontinence across Health Factors score**

| **Character** | **Low** | **Moderate (OR [95% CI])** | **P value** | **High (OR [95% CI])** | **P value** | **P for trend** | **P for interaction** |
| --- | --- | --- | --- | --- | --- | --- | --- |
| **Age group** |  |  |  |  |  |  | 0.24 |
| **20-40** | ref | 0.66(0.28,1.55) | 0.33 | 0.60(0.24,1.55) | 0.28 | 0.46 |  |
| **40-60** | ref | 0.79(0.48,1.30) | 0.34 | 0.67(0.41,1.10) | 0.11 | 0.12 |  |
| **≥60** | ref | 1.29(0.88,1.90) | 0.19 | 1.20(0.78,1.83) | 0.39 | 0.31 |  |
| **Sex** |  |  |  |  |  |  | 0.93 |
| **Female** | ref | 1.00(0.68,1.46) | 1 | 0.90(0.58,1.42) | 0.65 | 0.61 |  |
| **Male** | ref | 0.95(0.60,1.51) | 0.82 | 0.77(0.46,1.26) | 0.28 | 0.24 |  |
| **Ethnic** |  |  |  |  |  |  | 0.4 |
| **White** | ref | 0.90(0.62,1.30) | 0.57 | 0.78(0.53,1.16) | 0.22 | 0.21 |  |
| **Black** | ref | 1.62(0.83,3.16) | 0.15 | 1.31(0.68,2.53) | 0.41 | 0.44 |  |
| **Other** | ref | 1.07(0.51,2.24) | 0.86 | 0.89(0.42,1.89) | 0.76 | 0.67 |  |
| **Marital status** |  |  |  |  |  |  | 0.34 |
| **Married/With partner** | ref | 1.17(0.82,1.67) | 0.38 | 0.99(0.65,1.53) | 0.97 | 0.82 |  |
| **Alone** | ref | 0.78(0.44,1.38) | 0.37 | 0.68(0.41,1.14) | 0.14 | 0.15 |  |
| **Educational level** |  |  |  |  |  |  | 0.3 |
| **≤Highschool** | ref | 1.14(0.77,1.69) | 0.51 | 0.99(0.62,1.56) | 0.95 | 0.92 |  |
| **>Highschool** | ref | 0.85(0.55,1.32) | 0.46 | 0.75(0.45,1.24) | 0.25 | 0.26 |  |
| **Family size** |  |  |  |  |  |  | 0.39 |
| **1-3** | ref | 0.99(0.68,1.43) | 0.94 | 0.79(0.54,1.15) | 0.21 | 0.17 |  |
| **>3** | ref | 1.00(0.51,1.95) | 1 | 1.09(0.54,2.20) | 0.8 | 0.76 |  |
| **Annual family income** |  |  |  |  |  |  | 0.35 |
| **Under $20,000** | ref | 0.80(0.43,1.49) | 0.46 | 0.82(0.41,1.64) | 0.57 | 0.59 |  |
| **$20,000 - $35,000** | ref | 1.26(0.79,1.99) | 0.32 | 0.96(0.49,1.87) | 0.9 | 0.81 |  |
| **$35,000 - $75,000** | ref | 0.64(0.39,1.03) | 0.06 | 0.58(0.32,1.05) | 0.07 | 0.09 |  |
| **Over $75,000** | ref | 1.68(0.78, 3.65) | 0.18 | 1.25(0.62, 2.53) | 0.52 | 0.98 |  |
| **Smoking** |  |  |  |  |  |  | 0.21 |
| **No** | ref | 0.81(0.54,1.23) | 0.31 | 0.84(0.47,1.49) | 0.53 | 0.59 |  |
| **Yes** | ref | 1.21(0.81,1.79) | 0.34 | 0.86(0.57,1.29) | 0.45 | 0.27 |  |
| **Drinking** |  |  |  |  |  |  | 0.04 |
| **No** | ref | 1.20(0.51,2.82) | 0.67 | 1.61(0.60,4.33) | 0.33 | 0.32 |  |
| **Yes** | ref | 0.96(0.68,1.35) | 0.79 | 0.77(0.54,1.09) | 0.14 | 0.11 |  |
| **Weight status** |  |  |  |  |  |  | 0.2 |
| **Overweight** | ref | 1.22(0.53,2.81) | 0.64 | 1.04(0.42,2.57) | 0.92 | 0.82 |  |
| **Obese** | ref | 1.04(0.67,1.62) | 0.86 | 0.27(0.06,1.30) | 0.1 | 0.62 |  |
| **PHQ-9 (cut-off by 5)** |  |  |  |  |  |  | 0.47 |
| **No** | ref | 1.15(0.79,1.67) | 0.45 | 1.01(0.69,1.48) | 0.97 | 0.86 |  |
| **Yes** | ref | 0.78(0.48,1.28) | 0.32 | 0.66(0.40,1.09) | 0.1 | 0.1 |  |
| **Vigorous physical activity** |  |  |  |  |  |  | 0.03 |
| **No** | ref | 1.21(0.84,1.72) | 0.29 | 1.06(0.76,1.46) | 0.74 | 0.83 |  |
| **Yes** | ref | 0.54(0.27, 1.08) | 0.08 | 0.50(0.25, 0.98) | 0.04 | 0.09 |  |

Adjusted for age, sex, ehtnic, marital status, educational level, family size, annual family income, drinking, and PHQ-9

LE8: life’s essential 8; PHQ-9: 9-question Patient Health Questionnaire; BMI: Body Mass Index; OR: odds ratios; CI: confidence interval;

**Supplementary table 13. Stratified analysis of chronic constipation death across LE8 score**

| **Character** | **Low** | **Moderate (OR [95% CI])** | **P value** | **High (OR [95% CI])** | **P value** | **P for trend** | **P for interaction** |
| --- | --- | --- | --- | --- | --- | --- | --- |
| **Age group** |  |  |  |  |  |  | 0.46 |
| **20-40** | ref | 0.40(0.18, 0.90) | 0.03 | 0.00(0.00, 0.00) | <0.0001 | 0.11 |  |
| **40-60** | ref | 0.63(0.22, 1.78) | 0.38 | 0.04(0.00, 0.55) | 0.02 | 0.05 |  |
| **≥60** | ref | 0.50(0.24,1.07) | 0.07 | 0.18(0.09,0.38) | <0.0001 | <0.0001 |  |
| **Sex** |  |  |  |  |  |  | 0.74 |
| **Female** | ref | 0.52(0.27,1.01) | 0.05 | 0.19(0.08,0.46) | <0.001 | <0.001 |  |
| **Male** | ref | 0.68(0.20, 2.23) | 0.52 | 0.02(0.00, 0.24) | 0.002 | 0.04 |  |
| **Ethnic** |  |  |  |  |  |  | 0.78 |
| **White** | ref | 0.43(0.22,0.86) | 0.02 | 0.11(0.05,0.25) | <0.0001 | <0.0001 |  |
| **Black** | ref | 0.53(0.18,1.55) | 0.25 | 0.34(0.04,3.20) | 0.35 | 0.24 |  |
| **Other** | ref | 0.25(0.03, 2.39) | 0.23 | 0.26(0.01, 5.99) | 0.4 | 0.32 |  |
| **Marital status** |  |  |  |  |  |  | 0.95 |
| **Married/With partner** | ref | 0.63(0.30,1.32) | 0.22 | 0.21(0.07,0.60) | 0.004 | 0.002 |  |
| **Alone** | ref | 0.46(0.18,1.19) | 0.11 | 0.10(0.02,0.43) | 0.002 | 0.005 |  |
| **Educational level** |  |  |  |  |  |  | 0.7 |
| **≤Highschool** | ref | 0.65(0.31,1.36) | 0.25 | 0.31(0.07,1.35) | 0.12 | 0.09 |  |
| **>Highschool** | ref | 0.55(0.24,1.27) | 0.16 | 0.18(0.06,0.54) | 0.002 | 0.003 |  |
| **Family size** |  |  |  |  |  |  | 0.71 |
| **1-3** | ref | 0.60(0.31,1.16) | 0.13 | 0.22(0.09,0.53) | <0.001 | 0.003 |  |
| **>3** | ref | 0.15(0.04, 0.49) | 0.002 | 0.01(0.00, 0.20) | 0.004 | <0.0001 |  |
| **Annual family income** |  |  |  |  |  |  | 0.34 |
| **Under $20,000** | ref | 0.56(0.23,1.39) | 0.21 | 0.34(0.08,1.55) | 0.16 | 0.13 |  |
| **$20,000 - $35,000** | ref | 0.37(0.14,1.00) | 0.05 | 0.15(0.03,0.83) | 0.03 | 0.01 |  |
| **$35,000 - $75,000** | ref | 25.14(2.00,316.36) | 0.01 | 0.51(0.06, 4.40) | 0.54 | 0.3 |  |
| **Over $75,000** | ref | 1.33(0.16,11.27) | 0.79 | 0.08(0.00, 1.55) | 0.1 | 0.02 |  |
| **Smoking** |  |  |  |  |  |  | 0.31 |
| **No** | ref | 0.66(0.25,1.72) | 0.39 | 0.32(0.09,1.08) | 0.07 | 0.09 |  |
| **Yes** | ref | 0.46(0.19,1.15) | 0.1 | 0.00(0.00,0.00) | <0.0001 | 0.02 |  |
| **Drinking** |  |  |  |  |  |  | 0.39 |
| **No** | ref | 0.43(0.11, 1.72) | 0.23 | 0.45(0.07, 2.83) | 0.4 | 0.25 |  |
| **Yes** | ref | 0.55(0.27,1.13) | 0.11 | 0.15(0.05,0.46) | <0.001 | 0.002 |  |
| **Weight status** |  |  |  |  |  |  | 0.91 |
| **Under/Normal weight** | ref | 0.35(0.08,1.66) | 0.19 | 0.08(0.01,0.47) | 0.01 | 0.01 |  |
| **Overweight** | ref | 0.57(0.20, 1.62) | 0.29 | 0.15(0.04, 0.60) | 0.01 | 0.02 |  |
| **Obese** | ref | 0.24(0.09, 0.63) | 0.004 | 0.00(0.00, 0.00) | <0.0001 | 0.004 |  |
| **PHQ-9 (cut-off by 5)** |  |  |  |  |  |  | 0.58 |
| **No** | ref | 0.83(0.41,1.71) | 0.62 | 0.26(0.11,0.65) | 0.004 | 0.002 |  |
| **Yes** | ref | 0.39(0.10,1.46) | 0.16 | 0.00(0.00,0.00) | <0.0001 | 0.14 |  |
| **Vigorous physical activity** |  |  |  |  |  |  | 0.57 |
| **No** | ref | 0.53(0.28,1.00) | 0.05 | 0.22(0.10,0.47) | <0.0001 | <0.001 |  |
| **Yes** | ref | 0.28(0.03, 2.48) | 0.25 | 0.02(0.00, 0.50) | 0.02 | 0.01 |  |

Adjusted for age, sex, ehtnic, marital status, educational level, family size, annual family income, drinking, and PHQ-9

LE8: life’s essential 8; PHQ-9: 9-question Patient Health Questionnaire; BMI: Body Mass Index; OR: odds ratios; CI: confidence interval;

**Supplementary table 14. Stratified analysis of chronic diarrhea death across LE8 score**

| **Character** | **Low** | **Moderate (OR [95% CI])** | **P value** | **High (OR [95% CI])** | **P value** | **P for trend** | **P for interaction** |
| --- | --- | --- | --- | --- | --- | --- | --- |
| **Age group** |  |  |  |  |  |  | 0.1 |
| **20-40** | ref | 3.39(0.25,46.16) | 0.36 | 2.47(0.10,62.99) | 0.58 | 0.49 |  |
| **40-60** | ref | 0.58(0.18, 1.81) | 0.35 | 0.11(0.01, 0.81) | 0.03 | 0.22 |  |
| **≥60** | ref | 0.80(0.46,1.40) | 0.43 | 0.32(0.11,0.92) | 0.03 | 0.07 |  |
| **Sex** |  |  |  |  |  |  | 0.24 |
| **Female** | ref | 1.04(0.54,2.01) | 0.91 | 0.31(0.09,1.03) | 0.06 | 0.44 |  |
| **Male** | ref | 0.57(0.20,1.62) | 0.29 | 0.25(0.06,1.07) | 0.06 | 0.14 |  |
| **Ethnic** |  |  |  |  |  |  | 0.35 |
| **White** | ref | 0.76(0.34,1.73) | 0.52 | 0.22(0.06,0.76) | 0.02 | 0.13 |  |
| **Black** | ref | 0.47(0.20,1.07) | 0.07 | 0.40(0.05,3.49) | 0.41 | 0.1 |  |
| **Other** | ref | 10.51(1.32, 83.49) | 0.03 | 6.26(0.39,101.56) | 0.2 | 0.05 |  |
| **Marital status** |  |  |  |  |  |  | 0.63 |
| **Married/With partner** | ref | 1.10(0.49,2.47) | 0.82 | 0.55(0.17,1.78) | 0.32 | 0.67 |  |
| **Alone** | ref | 0.58(0.26,1.28) | 0.18 | 0.11(0.02,0.64) | 0.01 | 0.03 |  |
| **Educational level** |  |  |  |  |  |  | 0.24 |
| **≤Highschool** | ref | 0.86(0.40,1.84) | 0.7 | 0.64(0.22,1.85) | 0.41 | 0.57 |  |
| **>Highschool** | ref | 0.87(0.22,3.48) | 0.85 | 0.14(0.01,1.40) | 0.1 | 0.18 |  |
| **Family size** |  |  |  |  |  |  | 0.8 |
| **1-3** | ref | 0.67(0.32,1.39) | 0.28 | 0.23(0.07,0.73) | 0.01 | 0.06 |  |
| **>3** | ref | 1.50(0.48,4.73) | 0.49 | 0.39(0.06,2.57) | 0.33 | 0.93 |  |
| **Annual family income** |  |  |  |  |  |  | 0.08 |
| **Under $20,000** | ref | 0.77(0.40,1.50) | 0.44 | 0.56(0.27,1.16) | 0.12 | 0.23 |  |
| **$20,000 - $35,000** | ref | 0.33(0.13,0.85) | 0.02 | 0.10(0.01,0.70) | 0.02 | 0.01 |  |
| **$35,000 - $75,000** | ref | 3.48(0.90,13.55) | 0.07 | 1.06(0.13, 8.58) | 0.96 | 0.25 |  |
| **Over $75,000** | ref | 0.01(0.00, 0.55) | 0.02 | 0.01(0.00, 0.13) | <0.001 | 0.01 |  |
| **Smoking** |  |  |  |  |  |  | 0.29 |
| **No** | ref | 1.43(0.60,3.42) | 0.42 | 0.39(0.11,1.36) | 0.14 | 0.28 |  |
| **Yes** | ref | 0.61(0.27,1.36) | 0.23 | 0.29(0.06,1.43) | 0.13 | 0.15 |  |
| **Drinking** |  |  |  |  |  |  | 0.38 |
| **No** | ref | 1.38(0.48, 3.95) | 0.55 | 2.73(0.42,17.79) | 0.29 | 0.34 |  |
| **Yes** | ref | 0.77(0.39,1.51) | 0.44 | 0.18(0.05,0.65) | 0.01 | 0.07 |  |
| **Weight status** |  |  |  |  |  |  | 0.3 |
| **Under/Normal weight** | ref | 0.29(0.12, 0.71) | 0.01 | 0.26(0.05, 1.36) | 0.11 | 0.05 |  |
| **Overweight** | ref | 0.70(0.21,2.27) | 0.55 | 0.17(0.04,0.72) | 0.02 | 0.05 |  |
| **Obese** | ref | 0.78(0.32,1.93) | 0.59 | 0.00(0.00,0.00) | <0.0001 | 0.5 |  |
| **PHQ-9 (cut-off by 5)** |  |  |  |  |  |  | 0.58 |
| **No** | ref | 0.61(0.33,1.12) | 0.11 | 0.25(0.09,0.68) | 0.01 | 0.01 |  |
| **Yes** | ref | 1.07(0.20,5.84) | 0.93 | 0.00(0.00,0.00) | <0.0001 | 0.97 |  |
| **Vigorous physical activity** |  |  |  |  |  |  | 0.9 |
| **No** | ref | 0.70(0.36,1.37) | 0.3 | 0.28(0.07,1.13) | 0.07 | 0.16 |  |
| **Yes** | ref | 1.27(0.26,6.19) | 0.76 | 0.39(0.06,2.52) | 0.32 | 0.27 |  |

Adjusted for age, sex, ehtnic, marital status, educational level, family size, annual family income, drinking, and PHQ-9

LE8: life’s essential 8; PHQ-9: 9-question Patient Health Questionnaire; BMI: Body Mass Index; OR: odds ratios; CI: confidence interval;

**Supplementary table 15. Stratified analysis of fecal incontinence death across LE8 score**

| **Character** | **Low** | **Moderate (OR [95% CI])** | **P value** | **High (OR [95% CI])** | **P value** | **P for trend** | **P for interaction** |
| --- | --- | --- | --- | --- | --- | --- | --- |
| **Age group** |  |  |  |  |  |  | 0.17 |
| **40-60** | ref | 1.37(0.43, 4.41) | 0.59 | 0.00(0.00, 0.00) | <0.0001 | 0.72 |  |
| **≥60** | ref | 0.68(0.38,1.20) | 0.19 | 0.74(0.33,1.66) | 0.46 | 0.37 |  |
| **Sex** |  |  |  |  |  |  | 0.09 |
| **Female** | ref | 0.53(0.28,0.98) | **0.04** | 0.73(0.30,1.76) | 0.48 | 0.33 |  |
| **Male** | ref | 1.09(0.57, 2.07) | 0.8 | 0.34(0.06, 1.92) | 0.22 | 0.35 |  |
| **Ethnic** |  |  |  |  |  |  | 0.4 |
| **White** | ref | 0.74(0.40,1.37) | 0.34 | 0.73(0.32,1.68) | 0.46 | 0.41 |  |
| **Black** | ref | 0.38(0.18, 0.80) | **0.01** | 0.00(0.00, 0.00) | <0.0001 | **0.01** |  |
| **Other** | ref | 1.67(0.29, 9.69) | 0.57 | 0.60(0.02,15.03) | 0.75 | 0.78 |  |
| **Marital status** |  |  |  |  |  |  | 0.08 |
| **Married/With partner** | ref | 1.12(0.54,2.33) | 0.75 | 0.63(0.21,1.90) | 0.41 | 0.47 |  |
| **Alone** | ref | 0.43(0.24,0.77) | **0.005** | 0.44(0.18,1.10) | 0.08 | **0.02** |  |
| **Educational level** |  |  |  |  |  |  | 0.95 |
| **≤Highschool** | ref | 0.74(0.43,1.27) | 0.27 | 0.63(0.18,2.14) | 0.46 | 0.25 |  |
| **>Highschool** | ref | 0.59(0.24, 1.41) | 0.23 | 0.53(0.19, 1.51) | 0.24 | 0.35 |  |
| **Family size** |  |  |  |  |  |  | 0.6 |
| **1-3** | ref | 0.71(0.41,1.21) | 0.21 | 0.60(0.29,1.25) | 0.17 | 0.15 |  |
| **>3** | ref | 1.62(0.53, 4.97) | 0.4 | 10.68(0.29,395.12) | 0.2 | 0.35 |  |
| **Annual family income** |  |  |  |  |  |  | 0.87 |
| **Under $20,000** | ref | 0.78(0.36,1.66) | 0.51 | 0.93(0.25,3.44) | 0.91 | 0.54 |  |
| **$20,000 - $35,000** | ref | 0.48(0.21, 1.08) | 0.08 | 0.35(0.10, 1.23) | 0.1 | 0.08 |  |
| **$35,000 - $75,000** | ref | 1.17(0.47, 2.94) | 0.73 | 0.60(0.07, 5.15) | 0.64 | 0.72 |  |
| **Over $75,000** | ref | 1.20(0.21, 6.81) | 0.84 | 4.14(0.70, 24.45) | 0.12 | 0.06 |  |
| **Smoking** |  |  |  |  |  |  | 0.97 |
| **No** | ref | 0.91(0.35,2.35) | 0.85 | 1.11(0.41,2.98) | 0.84 | 0.84 |  |
| **Yes** | ref | 0.85(0.49,1.47) | 0.55 | 0.69(0.23,2.08) | 0.51 | 0.47 |  |
| **Drinking** |  |  |  |  |  |  | 0.59 |
| **No** | ref | 0.31(0.04, 2.80) | 0.3 | 1.74(0.25,12.17) | 0.57 | 0.35 |  |
| **Yes** | ref | 0.73(0.45,1.20) | 0.21 | 0.52(0.23,1.17) | 0.11 | 0.1 |  |
| **Weight status** |  |  |  |  |  |  | 0.98 |
| **Under/Normal weight** | ref | 0.70(0.12,4.21) | 0.7 | 0.53(0.09,3.22) | 0.49 | 0.46 |  |
| **Overweight** | ref | 0.52(0.20, 1.33) | 0.17 | 0.42(0.09, 2.06) | 0.29 | 0.3 |  |
| **Obese** | ref | 0.49(0.29, 0.83) | **0.01** | 0.00(0.00, 0.00) | <0.0001 | **0.01** |  |
| **PHQ-9 (cut-off by 5)** |  |  |  |  |  |  | 0.32 |
| **No** | ref | 0.58(0.30,1.11) | 0.1 | 0.53(0.22,1.28) | 0.16 | 0.21 |  |
| **Yes** | ref | 0.93(0.45,1.90) | 0.84 | 0.00(0.00,0.00) | <0.0001 | 0.55 |  |

Adjusted for age, sex, ehtnic, marital status, educational level, family size, annual family income, drinking, and PHQ-9

LE8: life’s essential 8; PHQ-9: 9-question Patient Health Questionnaire; BMI: Body Mass Index; OR: odds ratios; CI: confidence interval;

**Supplementary table 16. Stratified analysis of chronic constipation death across Health Behaviors score**

| **Character** | **Low** | **Moderate (OR [95% CI])** | **P value** | **High (OR [95% CI])** | **P value** | **P for trend** | **P for interaction** |
| --- | --- | --- | --- | --- | --- | --- | --- |
| **Age group** |  |  |  |  |  |  | 0.53 |
| **20-40** | ref | 1.39(0.27, 7.31) | 0.69 | 0.00(0.00, 0.00) | <0.0001 | 0.24 |  |
| **40-60** | ref | 0.73(0.27, 1.93) | 0.52 | 0.54(0.03, 9.31) | 0.67 | 0.64 |  |
| **≥60** | ref | 0.45(0.28,0.73) | 0.001 | 0.18(0.09,0.36) | <0.0001 | <0.0001 |  |
| **Sex** |  |  |  |  |  |  | 0.86 |
| **Female** | ref | 0.57(0.30,1.07) | 0.08 | 0.29(0.12,0.72) | 0.01 | 0.01 |  |
| **Male** | ref | 0.63(0.20, 1.97) | 0.42 | 0.12(0.03, 0.52) | 0.005 | 0.02 |  |
| **Ethnic** |  |  |  |  |  |  | 0.17 |
| **White** | ref | 0.57(0.29,1.10) | 0.09 | 0.17(0.07,0.38) | <0.0001 | <0.0001 |  |
| **Black** | ref | 0.30(0.08, 1.20) | 0.09 | 1.21(0.38, 3.85) | 0.75 | 0.78 |  |
| **Other** | ref | 0.07(0.01, 0.55) | 0.01 | 0.06(0.00, 1.39) | 0.08 | 0.11 |  |
| **Marital status** |  |  |  |  |  |  | 0.34 |
| **Married/With partner** | ref | 0.78(0.36,1.67) | 0.52 | 0.27(0.12,0.60) | 0.001 | 0.004 |  |
| **Alone** | ref | 0.44(0.15,1.24) | 0.12 | 0.35(0.12,1.06) | 0.06 | 0.07 |  |
| **Educational level** |  |  |  |  |  |  | 0.21 |
| **≤Highschool** | ref | 0.52(0.33,0.82) | 0.01 | 0.39(0.15,0.99) | 0.05 | 0.01 |  |
| **>Highschool** | ref | 0.73(0.29,1.86) | 0.51 | 0.20(0.05,0.83) | 0.03 | 0.02 |  |
| **Family size** |  |  |  |  |  |  | 0.41 |
| **1-3** | ref | 0.64(0.36,1.13) | 0.12 | 0.34(0.17,0.70) | 0.003 | 0.004 |  |
| **>3** | ref | 0.08(0.02, 0.44) | 0.003 | 0.01(0.00, 0.23) | 0.005 | <0.001 |  |
| **Annual family income** |  |  |  |  |  |  | 0.35 |
| **Under $20,000** | ref | 0.65(0.26, 1.60) | 0.35 | 0.39(0.08, 1.80) | 0.23 | 0.19 |  |
| **$20,000 - $35,000** | ref | 0.43(0.17,1.11) | 0.08 | 0.36(0.12,1.07) | 0.07 | 0.06 |  |
| **$35,000 - $75,000** | ref | 1.99(0.37,10.53) | 0.42 | 0.28(0.05, 1.69) | 0.17 | 0.01 |  |
| **Over $75,000** | ref | 0.54(0.15, 1.97) | 0.35 | 0.02(0.00, 0.11) | <0.0001 | <0.001 |  |
| **Smoking** |  |  |  |  |  |  | 0.15 |
| **No** | ref | 0.64(0.25,1.61) | 0.34 | 0.52(0.16,1.68) | 0.27 | 0.3 |  |
| **Yes** | ref | 0.49(0.20,1.18) | 0.11 | 0.03(0.01,0.17) | <0.0001 | 0.002 |  |
| **Drinking** |  |  |  |  |  |  | 0.28 |
| **No** | ref | 0.41(0.18, 0.95) | 0.04 | 0.48(0.07, 3.41) | 0.46 | 0.18 |  |
| **Yes** | ref | 0.61(0.32,1.18) | 0.14 | 0.27(0.12,0.58) | <0.001 | 0.002 |  |
| **Weight status** |  |  |  |  |  |  | 0.84 |
| **Under/Normal weight** | ref | 0.96(0.42, 2.20) | 0.92 | 0.36(0.13, 1.05) | 0.06 | 0.08 |  |
| **Overweight** | ref | 0.80(0.39, 1.63) | 0.54 | 0.36(0.08, 1.62) | 0.18 | 0.15 |  |
| **Obese** | ref | 0.60(0.17, 2.05) | 0.41 | 0.23(0.05, 1.09) | 0.06 | 0.16 |  |
| **PHQ-9 (cut-off by 5)** |  |  |  |  |  |  | 0.13 |
| **No** | ref | 0.58(0.39,0.85) | 0.01 | 0.26(0.14,0.51) | <0.0001 | <0.0001 |  |
| **Yes** | ref | 0.72(0.16,3.21) | 0.67 | 1.76(0.56,5.55) | 0.33 | 0.98 |  |
| **Vigorous physical activity** |  |  |  |  |  |  | 0.89 |
| **No** | ref | 0.54(0.35,0.83) | 0.004 | 0.23(0.13,0.42) | <0.0001 | <0.0001 |  |
| **Yes** | ref | 1.99(0.13,30.36) | 0.62 | 1.08(0.02,54.97) | 0.97 | 0.82 |  |

Adjusted for age, sex, ehtnic, marital status, educational level, family size, annual family income, drinking, and PHQ-9

LE8: life’s essential 8; PHQ-9: 9-question Patient Health Questionnaire; BMI: Body Mass Index; OR: odds ratios; CI: confidence interval;

**Supplementary table 17. Stratified analysis of chronic diarrhea death across Health Behaviors score**

| **Character** | **Low** | **Moderate (OR [95% CI])** | **P value** | **High (OR [95% CI])** | **P value** | **P for trend** | **P for interaction** |
| --- | --- | --- | --- | --- | --- | --- | --- |
| **Age group** |  |  |  |  |  |  | < 0.001 |
| **40-60** | ref | 0.48(0.15, 1.50) | 0.21 | 0.04(0.01, 0.14) | <0.0001 | 0.03 |  |
| **≥60** | ref | 1.06(0.51,2.21) | 0.87 | 0.68(0.30,1.52) | 0.34 | 0.29 |  |
| **Sex** |  |  |  |  |  |  | 0.69 |
| **Female** | ref | 0.74(0.33,1.66) | 0.46 | 0.30(0.14,0.64) | 0.002 | 0.01 |  |
| **Male** | ref | 1.08(0.44,2.65) | 0.86 | 0.78(0.29,2.14) | 0.63 | 0.67 |  |
| **Ethnic** |  |  |  |  |  |  | 0.64 |
| **White** | ref | 0.86(0.41,1.82) | 0.7 | 0.44(0.18,1.05) | 0.07 | 0.07 |  |
| **Black** | ref | 0.62(0.21,1.82) | 0.39 | 0.41(0.13,1.29) | 0.13 | 0.18 |  |
| **Other** | ref | 4.93(0.49,49.82) | 0.18 | 3.37(0.25,46.16) | 0.36 | 0.24 |  |
| **Marital status** |  |  |  |  |  |  | 0.57 |
| **Married/With partner** | ref | 1.12(0.36,3.45) | 0.85 | 0.51(0.15,1.79) | 0.29 | 0.28 |  |
| **Alone** | ref | 0.61(0.31,1.22) | 0.16 | 0.40(0.16,1.00) | 0.05 | 0.05 |  |
| **Educational level** |  |  |  |  |  |  | 0.31 |
| **≤Highschool** | ref | 0.78(0.37,1.66) | 0.52 | 0.49(0.23,1.06) | 0.07 | 0.12 |  |
| **>Highschool** | ref | 1.68(0.68,4.16) | 0.26 | 0.50(0.13,1.91) | 0.31 | 0.16 |  |
| **Family size** |  |  |  |  |  |  | 0.23 |
| **1-3** | ref | 0.68(0.34,1.36) | 0.27 | 0.36(0.17,0.75) | 0.01 | 0.01 |  |
| **>3** | ref | 1.65(0.61,4.43) | 0.32 | 1.00(0.38,2.68) | 1 | 0.53 |  |
| **Annual family income** |  |  |  |  |  |  | 0.05 |
| **Under $20,000** | ref | 1.10(0.51,2.38) | 0.81 | 0.51(0.21,1.27) | 0.15 | 0.21 |  |
| **$20,000 - $35,000** | ref | 0.21(0.09,0.51) | <0.001 | 0.16(0.05,0.58) | 0.01 | 0.02 |  |
| **$35,000 - $75,000** | ref | 1.73(0.60, 4.95) | 0.31 | 0.60(0.15, 2.40) | 0.47 | 0.66 |  |
| **Over $75,000** | ref | 0.26(0.01, 5.73) | 0.39 | 0.51(0.05, 4.87) | 0.56 | 0.82 |  |
| **Smoking** |  |  |  |  |  |  | 0.08 |
| **No** | ref | 2.96(0.66,13.25) | 0.16 | 1.28(0.29, 5.76) | 0.75 | 0.1 |  |
| **Yes** | ref | 0.64(0.28,1.46) | 0.28 | 0.37(0.14,0.99) | 0.05 | 0.1 |  |
| **Drinking** |  |  |  |  |  |  | 0.51 |
| **No** | ref | 1.70(0.40,7.24) | 0.47 | 1.56(0.39,6.22) | 0.53 | 0.56 |  |
| **Yes** | ref | 0.83(0.44,1.57) | 0.57 | 0.38(0.17,0.84) | 0.02 | 0.02 |  |
| **Weight status** |  |  |  |  |  |  | 0.01 |
| **Under/Normal weight** | ref | 0.17(0.07, 0.43) | <0.001 | 0.35(0.10, 1.24) | 0.1 | 0.13 |  |
| **Overweight** | ref | 1.32(0.48,3.62) | 0.58 | 0.38(0.13,1.17) | 0.09 | 0.06 |  |
| **Obese** | ref | 1.22(0.45,3.29) | 0.7 | 0.85(0.25,2.85) | 0.79 | 0.85 |  |
| **PHQ-9 (cut-off by 5)** |  |  |  |  |  |  | 0.94 |
| **No** | ref | 0.83(0.41,1.67) | 0.6 | 0.40(0.17,0.97) | 0.04 | 0.03 |  |
| **Yes** | ref | 0.75(0.25,2.28) | 0.61 | 0.65(0.21,2.04) | 0.46 | 0.47 |  |
| **Vigorous physical activity** |  |  |  |  |  |  | 0.31 |
| **No** | ref | 0.73(0.40,1.33) | 0.3 | 0.32(0.15,0.68) | 0.003 | 0.01 |  |
| **Yes** | ref | 2.09(0.52,8.39) | 0.3 | 1.30(0.25,6.81) | 0.76 | 0.95 |  |

Adjusted for age, sex, ehtnic, marital status, educational level, family size, annual family income, drinking, and PHQ-9

LE8: life’s essential 8; PHQ-9: 9-question Patient Health Questionnaire; BMI: Body Mass Index; OR: odds ratios; CI: confidence interval;

**Supplementary table 18. Stratified analysis of fecal incontinence death across Health Behaviors score**

| **Character** | **Low** | **Moderate (OR [95% CI])** | **P value** | **High (OR [95% CI])** | **P value** | **P for trend** | **P for interaction** |
| --- | --- | --- | --- | --- | --- | --- | --- |
| **Age group** |  |  |  |  |  |  | 0.26 |
| **40-60** | ref | 0.47(0.13, 1.69) | 0.25 | 0.73(0.21, 2.55) | 0.62 | 0.48 |  |
| **≥60** | ref | 0.70(0.49,1.01) | 0.05 | 0.48(0.29,0.78) | 0.003 | 0.003 |  |
| **Sex** |  |  |  |  |  |  | 0.03 |
| **Female** | ref | 0.42(0.23,0.78) | 0.01 | 0.31(0.17,0.57) | <0.001 | <0.001 |  |
| **Male** | ref | 1.34(0.64, 2.83) | 0.44 | 1.02(0.46, 2.25) | 0.96 | 0.93 |  |
| **Ethnic** |  |  |  |  |  |  | 0.03 |
| **White** | ref | 0.51(0.32,0.79) | 0.003 | 0.47(0.27,0.80) | 0.01 | 0.01 |  |
| **Black** | ref | 0.63(0.30, 1.29) | 0.2 | 0.00(0.00, 0.00) | <0.0001 | 0.06 |  |
| **Other** | ref | 4.50(0.72,28.33) | 0.11 | 0.52(0.07, 3.68) | 0.52 | 0.54 |  |
| **Marital status** |  |  |  |  |  |  | 0.58 |
| **Married/With partner** | ref | 0.75(0.38,1.48) | 0.4 | 0.48(0.26,0.86) | 0.01 | 0.01 |  |
| **Alone** | ref | 0.55(0.30,0.99) | 0.05 | 0.42(0.22,0.81) | 0.01 | 0.01 |  |
| **Educational level** |  |  |  |  |  |  | 0.51 |
| **≤Highschool** | ref | 0.60(0.37,0.97) | 0.04 | 0.45(0.26,0.78) | 0.005 | 0.002 |  |
| **>Highschool** | ref | 0.99(0.42, 2.32) | 0.98 | 0.64(0.21, 1.90) | 0.42 | 0.34 |  |
| **Family size** |  |  |  |  |  |  | 0.53 |
| **1-3** | ref | 0.66(0.42,1.05) | 0.08 | 0.45(0.30,0.67) | <0.001 | <0.001 |  |
| **>3** | ref | 1.14(0.29, 4.52) | 0.85 | 2.05(0.50, 8.37) | 0.32 | 0.35 |  |
| **Annual family income** |  |  |  |  |  |  | 0.9 |
| **Under $20,000** | ref | 0.74(0.35,1.55) | 0.42 | 0.48(0.20,1.19) | 0.11 | 0.1 |  |
| **$20,000 - $35,000** | ref | 0.57(0.25, 1.29) | 0.18 | 0.51(0.14, 1.84) | 0.31 | 0.31 |  |
| **$35,000 - $75,000** | ref | 0.96(0.45, 2.08) | 0.92 | 0.62(0.12, 3.13) | 0.56 | 0.53 |  |
| **Over $75,000** | ref | 0.78(0.15, 3.93) | 0.76 | 0.58(0.17, 1.94) | 0.38 | 0.42 |  |
| **Smoking** |  |  |  |  |  |  | 0.32 |
| **No** | ref | 2.03(0.86,4.81) | 0.11 | 1.69(0.65,4.40) | 0.28 | 0.79 |  |
| **Yes** | ref | 0.66(0.38,1.17) | 0.15 | 0.50(0.16,1.53) | 0.22 | 0.16 |  |
| **Drinking** |  |  |  |  |  |  | 0.36 |
| **No** | ref | 0.24(0.02, 3.62) | 0.3 | 1.75(0.30,10.35) | 0.54 | 0.16 |  |
| **Yes** | ref | 0.68(0.45,1.03) | 0.07 | 0.48(0.30,0.77) | 0.002 | 0.002 |  |
| **Weight status** |  |  |  |  |  |  | 0.14 |
| **Under/Normal weight** | ref | 0.59(0.27,1.31) | 0.2 | 0.48(0.17,1.34) | 0.16 | 0.1 |  |
| **Overweight** | ref | 0.94(0.41, 2.17) | 0.89 | 0.39(0.13, 1.14) | 0.08 | 0.04 |  |
| **Obese** | ref | 0.57(0.31, 1.04) | 0.07 | 0.76(0.35, 1.66) | 0.5 | 0.53 |  |
| **PHQ-9 (cut-off by 5)** |  |  |  |  |  |  | 0.12 |
| **No** | ref | 0.80(0.45,1.40) | 0.43 | 0.71(0.40,1.25) | 0.23 | 0.27 |  |
| **Yes** | ref | 0.64(0.26,1.60) | 0.34 | 0.21(0.10,0.44) | <0.0001 | 0.002 |  |
| **Vigorous physical activity** |  |  |  |  |  |  | 0.26 |
| **No** | ref | 0.63(0.38,1.03) | 0.06 | 0.43(0.28,0.68) | <0.001 | <0.001 |  |
| **Yes** | ref | 2.08(0.20,21.63) | 0.54 | 1.57(0.13,19.10) | 0.72 | 0.97 |  |

Adjusted for age, sex, ehtnic, marital status, educational level, family size, annual family income, drinking, and PHQ-9

LE8: life’s essential 8; PHQ-9: 9-question Patient Health Questionnaire; BMI: Body Mass Index; OR: odds ratios; CI: confidence interval;

**Supplementary table 19. Stratified analysis of chronic constipation death across Health Factors score**

| **Character** | **Low** | **Moderate (OR [95% CI])** | **P value** | **High (OR [95% CI])** | **P value** | **P for trend** | **P for interaction** |
| --- | --- | --- | --- | --- | --- | --- | --- |
| **Age group** |  |  |  |  |  |  | 0.53 |
| **20-40** | ref | 1.39(0.27, 7.31) | 0.69 | 0.00(0.00, 0.00) | <0.0001 | 0.24 |  |
| **40-60** | ref | 0.73(0.27, 1.93) | 0.52 | 0.54(0.03, 9.31) | 0.67 | 0.64 |  |
| **≥60** | ref | 0.45(0.28,0.73) | 0.001 | 0.18(0.09,0.36) | <0.0001 | <0.0001 |  |
| **Sex** |  |  |  |  |  |  | 0.86 |
| **Female** | ref | 0.57(0.30,1.07) | 0.08 | 0.29(0.12,0.72) | 0.01 | 0.01 |  |
| **Male** | ref | 0.63(0.20, 1.97) | 0.42 | 0.12(0.03, 0.52) | 0.005 | 0.02 |  |
| **Ethnic** |  |  |  |  |  |  | 0.17 |
| **White** | ref | 0.57(0.29,1.10) | 0.09 | 0.17(0.07,0.38) | <0.0001 | <0.0001 |  |
| **Black** | ref | 0.30(0.08, 1.20) | 0.09 | 1.21(0.38, 3.85) | 0.75 | 0.78 |  |
| **Other** | ref | 0.07(0.01, 0.55) | 0.01 | 0.06(0.00, 1.39) | 0.08 | 0.11 |  |
| **Marital status** |  |  |  |  |  |  | 0.34 |
| **Married/With partner** | ref | 0.78(0.36,1.67) | 0.52 | 0.27(0.12,0.60) | 0.001 | 0.004 |  |
| **Alone** | ref | 0.44(0.15,1.24) | 0.12 | 0.35(0.12,1.06) | 0.06 | 0.07 |  |
| **Educational level** |  |  |  |  |  |  | 0.21 |
| **≤Highschool** | ref | 0.52(0.33,0.82) | 0.01 | 0.39(0.15,0.99) | 0.05 | 0.01 |  |
| **>Highschool** | ref | 0.73(0.29,1.86) | 0.51 | 0.20(0.05,0.83) | 0.03 | 0.02 |  |
| **Family size** |  |  |  |  |  |  | 0.41 |
| **1-3** | ref | 0.64(0.36,1.13) | 0.12 | 0.34(0.17,0.70) | 0.003 | 0.004 |  |
| **>3** | ref | 0.08(0.02, 0.44) | 0.003 | 0.01(0.00, 0.23) | 0.005 | <0.001 |  |
| **Annual family income** |  |  |  |  |  |  | 0.35 |
| **Under $20,000** | ref | 0.65(0.26, 1.60) | 0.35 | 0.39(0.08, 1.80) | 0.23 | 0.19 |  |
| **$20,000 - $35,000** | ref | 0.43(0.17,1.11) | 0.08 | 0.36(0.12,1.07) | 0.07 | 0.06 |  |
| **$35,000 - $75,000** | ref | 1.99(0.37,10.53) | 0.42 | 0.28(0.05, 1.69) | 0.17 | 0.01 |  |
| **Over $75,000** | ref | 0.54(0.15, 1.97) | 0.35 | 0.02(0.00, 0.11) | <0.0001 | <0.001 |  |
| **Smoking** |  |  |  |  |  |  | 0.15 |
| **No** | ref | 0.64(0.25,1.61) | 0.34 | 0.52(0.16,1.68) | 0.27 | 0.3 |  |
| **Yes** | ref | 0.49(0.20,1.18) | 0.11 | 0.03(0.01,0.17) | <0.0001 | 0.002 |  |
| **Drinking** |  |  |  |  |  |  | 0.28 |
| **No** | ref | 0.41(0.18, 0.95) | 0.04 | 0.48(0.07, 3.41) | 0.46 | 0.18 |  |
| **Yes** | ref | 0.61(0.32,1.18) | 0.14 | 0.27(0.12,0.58) | <0.001 | 0.002 |  |
| **Weight status** |  |  |  |  |  |  | 0.84 |
| **Under/Normal weight** | ref | 0.96(0.42, 2.20) | 0.92 | 0.36(0.13, 1.05) | 0.06 | 0.08 |  |
| **Overweight** | ref | 0.80(0.39, 1.63) | 0.54 | 0.36(0.08, 1.62) | 0.18 | 0.15 |  |
| **Obese** | ref | 0.60(0.17, 2.05) | 0.41 | 0.23(0.05, 1.09) | 0.06 | 0.16 |  |
| **PHQ-9 (cut-off by 5)** |  |  |  |  |  |  | 0.13 |
| **No** | ref | 0.58(0.39,0.85) | 0.01 | 0.26(0.14,0.51) | <0.0001 | <0.0001 |  |
| **Yes** | ref | 0.72(0.16,3.21) | 0.67 | 1.76(0.56,5.55) | 0.33 | 0.98 |  |
| **Vigorous physical activity** |  |  |  |  |  |  | 0.89 |
| **No** | ref | 0.54(0.35,0.83) | 0.004 | 0.23(0.13,0.42) | <0.0001 | <0.0001 |  |
| **Yes** | ref | 1.99(0.13,30.36) | 0.62 | 1.08(0.02,54.97) | 0.97 | 0.82 |  |

Adjusted for age, sex, ehtnic, marital status, educational level, family size, annual family income, drinking, and PHQ-9

LE8: life’s essential 8; PHQ-9: 9-question Patient Health Questionnaire; BMI: Body Mass Index; OR: odds ratios; CI: confidence interval;

**Supplementary table 20. Stratified analysis of chronic diarrhea death across Health Factors score**

| **Character** | **Low** | **Moderate (OR [95% CI])** | **P value** | **High (OR [95% CI])** | **P value** | **P for trend** | **P for interaction** |
| --- | --- | --- | --- | --- | --- | --- | --- |
| **Age group** |  |  |  |  |  |  | < 0.001 |
| **40-60** | ref | 0.48(0.15, 1.50) | 0.21 | 0.04(0.01, 0.14) | <0.0001 | 0.03 |  |
| **≥60** | ref | 1.06(0.51,2.21) | 0.87 | 0.68(0.30,1.52) | 0.34 | 0.29 |  |
| **Sex** |  |  |  |  |  |  | 0.69 |
| **Female** | ref | 0.74(0.33,1.66) | 0.46 | 0.30(0.14,0.64) | 0.002 | 0.01 |  |
| **Male** | ref | 1.08(0.44,2.65) | 0.86 | 0.78(0.29,2.14) | 0.63 | 0.67 |  |
| **Ethnic** |  |  |  |  |  |  | 0.64 |
| **White** | ref | 0.86(0.41,1.82) | 0.7 | 0.44(0.18,1.05) | 0.07 | 0.07 |  |
| **Black** | ref | 0.62(0.21,1.82) | 0.39 | 0.41(0.13,1.29) | 0.13 | 0.18 |  |
| **Other** | ref | 4.93(0.49,49.82) | 0.18 | 3.37(0.25,46.16) | 0.36 | 0.24 |  |
| **Marital status** |  |  |  |  |  |  | 0.57 |
| **Married/With partner** | ref | 1.12(0.36,3.45) | 0.85 | 0.51(0.15,1.79) | 0.29 | 0.28 |  |
| **Alone** | ref | 0.61(0.31,1.22) | 0.16 | 0.40(0.16,1.00) | 0.05 | 0.05 |  |
| **Educational level** |  |  |  |  |  |  | 0.31 |
| **≤Highschool** | ref | 0.78(0.37,1.66) | 0.52 | 0.49(0.23,1.06) | 0.07 | 0.12 |  |
| **>Highschool** | ref | 1.68(0.68,4.16) | 0.26 | 0.50(0.13,1.91) | 0.31 | 0.16 |  |
| **Family size** |  |  |  |  |  |  | 0.23 |
| **1-3** | ref | 0.68(0.34,1.36) | 0.27 | 0.36(0.17,0.75) | 0.01 | 0.01 |  |
| **>3** | ref | 1.65(0.61,4.43) | 0.32 | 1.00(0.38,2.68) | 1 | 0.53 |  |
| **Annual family income** |  |  |  |  |  |  | 0.05 |
| **Under $20,000** | ref | 1.10(0.51,2.38) | 0.81 | 0.51(0.21,1.27) | 0.15 | 0.21 |  |
| **$20,000 - $35,000** | ref | 0.21(0.09,0.51) | <0.001 | 0.16(0.05,0.58) | 0.01 | 0.02 |  |
| **$35,000 - $75,000** | ref | 1.73(0.60, 4.95) | 0.31 | 0.60(0.15, 2.40) | 0.47 | 0.66 |  |
| **Over $75,000** | ref | 0.26(0.01, 5.73) | 0.39 | 0.51(0.05, 4.87) | 0.56 | 0.82 |  |
| **Smoking** |  |  |  |  |  |  | 0.08 |
| **No** | ref | 2.96(0.66,13.25) | 0.16 | 1.28(0.29, 5.76) | 0.75 | 0.1 |  |
| **Yes** | ref | 0.64(0.28,1.46) | 0.28 | 0.37(0.14,0.99) | 0.05 | 0.1 |  |
| **Drinking** |  |  |  |  |  |  | 0.51 |
| **No** | ref | 1.70(0.40,7.24) | 0.47 | 1.56(0.39,6.22) | 0.53 | 0.56 |  |
| **Yes** | ref | 0.83(0.44,1.57) | 0.57 | 0.38(0.17,0.84) | 0.02 | 0.02 |  |
| **Weight status** |  |  |  |  |  |  | 0.01 |
| **Under/Normal weight** | ref | 0.17(0.07, 0.43) | <0.001 | 0.35(0.10, 1.24) | 0.1 | 0.13 |  |
| **Overweight** | ref | 1.32(0.48,3.62) | 0.58 | 0.38(0.13,1.17) | 0.09 | 0.06 |  |
| **Obese** | ref | 1.22(0.45,3.29) | 0.7 | 0.85(0.25,2.85) | 0.79 | 0.85 |  |
| **PHQ-9 (cut-off by 5)** |  |  |  |  |  |  | 0.94 |
| **No** | ref | 0.83(0.41,1.67) | 0.6 | 0.40(0.17,0.97) | 0.04 | 0.03 |  |
| **Yes** | ref | 0.75(0.25,2.28) | 0.61 | 0.65(0.21,2.04) | 0.46 | 0.47 |  |
| **Vigorous physical activity** |  |  |  |  |  |  | 0.31 |
| **No** | ref | 0.73(0.40,1.33) | 0.3 | 0.32(0.15,0.68) | 0.003 | 0.01 |  |
| **Yes** | ref | 2.09(0.52,8.39) | 0.3 | 1.30(0.25,6.81) | 0.76 | 0.95 |  |

Adjusted for age, sex, ehtnic, marital status, educational level, family size, annual family income, drinking, and PHQ-9

LE8: life’s essential 8; PHQ-9: 9-question Patient Health Questionnaire; BMI: Body Mass Index; OR: odds ratios; CI: confidence interval;

**Supplementary table 21. Stratified analysis of fecal incontinence death across Health Factors score**

| **Character** | **Low** | **Moderate (OR [95% CI])** | **P value** | **High (OR [95% CI])** | **P value** | **P for trend** | **P for interaction** |
| --- | --- | --- | --- | --- | --- | --- | --- |
| **Age group** |  |  |  |  |  |  | 0.26 |
| **40-60** | ref | 0.47(0.13, 1.69) | 0.25 | 0.73(0.21, 2.55) | 0.62 | 0.48 |  |
| **≥60** | ref | 0.70(0.49,1.01) | 0.05 | 0.48(0.29,0.78) | 0.003 | 0.003 |  |
| **Sex** |  |  |  |  |  |  | 0.03 |
| **Female** | ref | 0.42(0.23,0.78) | 0.01 | 0.31(0.17,0.57) | <0.001 | <0.001 |  |
| **Male** | ref | 1.34(0.64, 2.83) | 0.44 | 1.02(0.46, 2.25) | 0.96 | 0.93 |  |
| **Ethnic** |  |  |  |  |  |  | 0.03 |
| **White** | ref | 0.51(0.32,0.79) | 0.003 | 0.47(0.27,0.80) | 0.01 | 0.01 |  |
| **Black** | ref | 0.63(0.30, 1.29) | 0.2 | 0.00(0.00, 0.00) | <0.0001 | 0.06 |  |
| **Other** | ref | 4.50(0.72,28.33) | 0.11 | 0.52(0.07, 3.68) | 0.52 | 0.54 |  |
| **Marital status** |  |  |  |  |  |  | 0.58 |
| **Married/With partner** | ref | 0.75(0.38,1.48) | 0.4 | 0.48(0.26,0.86) | 0.01 | 0.01 |  |
| **Alone** | ref | 0.55(0.30,0.99) | 0.05 | 0.42(0.22,0.81) | 0.01 | 0.01 |  |
| **Educational level** |  |  |  |  |  |  | 0.51 |
| **≤Highschool** | ref | 0.60(0.37,0.97) | 0.04 | 0.45(0.26,0.78) | 0.005 | 0.002 |  |
| **>Highschool** | ref | 0.99(0.42, 2.32) | 0.98 | 0.64(0.21, 1.90) | 0.42 | 0.34 |  |
| **Family size** |  |  |  |  |  |  | 0.53 |
| **1-3** | ref | 0.66(0.42,1.05) | 0.08 | 0.45(0.30,0.67) | <0.001 | <0.001 |  |
| **>3** | ref | 1.14(0.29, 4.52) | 0.85 | 2.05(0.50, 8.37) | 0.32 | 0.35 |  |
| **Annual family income** |  |  |  |  |  |  | 0.9 |
| **Under $20,000** | ref | 0.74(0.35,1.55) | 0.42 | 0.48(0.20,1.19) | 0.11 | 0.1 |  |
| **$20,000 - $35,000** | ref | 0.57(0.25, 1.29) | 0.18 | 0.51(0.14, 1.84) | 0.31 | 0.31 |  |
| **$35,000 - $75,000** | ref | 0.96(0.45, 2.08) | 0.92 | 0.62(0.12, 3.13) | 0.56 | 0.53 |  |
| **Over $75,000** | ref | 0.78(0.15, 3.93) | 0.76 | 0.58(0.17, 1.94) | 0.38 | 0.42 |  |
| **Smoking** |  |  |  |  |  |  | 0.32 |
| **No** | ref | 2.03(0.86,4.81) | 0.11 | 1.69(0.65,4.40) | 0.28 | 0.79 |  |
| **Yes** | ref | 0.66(0.38,1.17) | 0.15 | 0.50(0.16,1.53) | 0.22 | 0.16 |  |
| **Drinking** |  |  |  |  |  |  | 0.36 |
| **No** | ref | 0.24(0.02, 3.62) | 0.3 | 1.75(0.30,10.35) | 0.54 | 0.16 |  |
| **Yes** | ref | 0.68(0.45,1.03) | 0.07 | 0.48(0.30,0.77) | 0.002 | 0.002 |  |
| **Weight status** |  |  |  |  |  |  | 0.14 |
| **Under/Normal weight** | ref | 0.59(0.27,1.31) | 0.2 | 0.48(0.17,1.34) | 0.16 | 0.1 |  |
| **Overweight** | ref | 0.94(0.41, 2.17) | 0.89 | 0.39(0.13, 1.14) | 0.08 | 0.04 |  |
| **Obese** | ref | 0.57(0.31, 1.04) | 0.07 | 0.76(0.35, 1.66) | 0.5 | 0.53 |  |
| **PHQ-9 (cut-off by 5)** |  |  |  |  |  |  | 0.12 |
| **No** | ref | 0.80(0.45,1.40) | 0.43 | 0.71(0.40,1.25) | 0.23 | 0.27 |  |
| **Yes** | ref | 0.64(0.26,1.60) | 0.34 | 0.21(0.10,0.44) | <0.0001 | 0.002 |  |
| **Vigorous physical activity** |  |  |  |  |  |  | 0.26 |
| **No** | ref | 0.63(0.38,1.03) | 0.06 | 0.43(0.28,0.68) | <0.001 | <0.001 |  |
| **Yes** | ref | 2.08(0.20,21.63) | 0.54 | 1.57(0.13,19.10) | 0.72 | 0.97 |  |

Adjusted for age, sex, ehtnic, marital status, educational level, family size, annual family income, drinking, and PHQ-9

LE8: life’s essential 8; PHQ-9: 9-question Patient Health Questionnaire; BMI: Body Mass Index; OR: odds ratios; CI: confidence interval;

**Supplementary table 22. The association between Bowel Health, Fecal Incontinence and CVH after excluding participants taking gastrointestinal and psychotherapeutic medications**

|  | **LE8 classification** | | | | | | | |
| --- | --- | --- | --- | --- | --- | --- | --- | --- |
|  | Crude model | | Model 1 | | Model 2 | | Model 3 | |
| **Chronic constipation** | 95%CI | P | 95%CI | P | 95%CI | P | 95%CI | P |
| Low | ref |  | ref |  | ref |  |  |  |
| Moderate | 0.61(0.41,0.91) | 0.02 | 0.55(0.37,0.81) | 0.003 | 0.67(0.46,0.98) | 0.04 |  |  |
| High | 0.67(0.42,1.06) | 0.08 | 0.47(0.29,0.74) | 0.002 | 0.71(0.45,1.12) | 0.14 |  |  |
| P for trend |  | 0.31 |  | 0.01 |  | 0.35 |  |  |
| **Chronic diarrhea** |  | | | | | |  |  |
| Low | ref |  | ref |  | ref |  |  |  |
| Moderate | 0.84(0.61,1.14) | 0.25 | 0.85(0.62,1.16) | 0.29 | 0.92(0.68,1.26) | 0.60 |  |  |
| High | 0.38(0.25,0.57) | <0.0001 | 0.40(0.26,0.62) | <0.001 | 0.49(0.31,0.77) | 0.003 |  |  |
| P for trend |  | <0.0001 |  | <0.0001 |  | <0.001 |  |  |
| **Fecal incontinence** |  | | | | | |  |  |
| Low | ref |  | ref |  | ref |  |  |  |
| Moderate | 0.67(0.45,1.00) | 0.05 | 0.73(0.50,1.08) | 0.11 | 0.82(0.56,1.19) | 0.28 |  |  |
| High | 0.47(0.30,0.71) | <0.001 | 0.60(0.40,0.89) | 0.01 | 0.73(0.48,1.12) | 0.14 |  |  |
| P for trend |  | <0.001 |  | 0.02 |  | 0.18 |  |  |
|  | **Health behaviors classification** | | | | | | | |
| **Chronic constipation** | 95%CI | P | 95%CI | P | 95%CI | P | 95%CI | P |
| Low | ref |  | ref |  | ref |  | ref |  |
| Moderate | 0.69(0.52,0.91) | 0.01 | 0.69(0.53,0.91) | 0.01 | 0.82(0.64,1.07) | 0.14 | 0.84(0.64,1.09) | 0.18 |
| High | 0.56(0.40,0.77) | <0.001 | 0.53(0.39,0.73) | <0.001 | 0.71(0.52,0.99) | 0.04 | 0.70(0.51,0.97) | 0.03 |
| P for trend |  | 0.001 |  | <0.001 |  | 0.04 |  | 0.03 |
| **Chronic diarrhea** |  | | | | | | | |
| Low | ref |  | ref |  | ref |  | ref |  |
| Moderate | 0.98(0.72,1.34) | 0.90 | 0.97(0.70,1.33) | 0.83 | 1.07(0.77,1.48) | 0.68 | 1.04(0.75,1.45) | 0.80 |
| High | 0.75(0.50,1.14) | 0.17 | 0.74(0.48,1.13) | 0.16 | 0.90(0.57,1.40) | 0.62 | 0.90(0.57,1.41) | 0.63 |
| P for trend |  | 0.14 |  | 0.13 |  | 0.57 |  | 0.59 |
| **Fecal incontinence** |  | | | | | | | |
| Low | ref |  | ref |  | ref |  | ref |  |
| Moderate | 0.80(0.59,1.09) | 0.15 | 0.72(0.53,0.99) | 0.04 | 0.80(0.59,1.08) | 0.15 | 0.81(0.60,1.09) | 0.15 |
| High | 0.66(0.48,0.90) | 0.01 | 0.53(0.38,0.73) | <0.001 | 0.62(0.44,0.87) | 0.01 | 0.63(0.44,0.90) | 0.01 |
| P for trend |  | 0.01 |  | <0.001 |  | 0.01 |  | 0.01 |
|  | **Health factors classification** | | | | | | | |
| **Chronic constipation** | 95%CI | P | 95%CI | P | 95%CI | P | 95%CI | P |
| Low | ref |  | ref |  | ref |  | ref |  |
| Moderate | 1.03(0.68,1.56) | 0.89 | 0.97(0.62,1.50) | 0.87 | 1.06(0.68,1.67) | 0.78 | 1.07(0.69,1.68) | 0.75 |
| High | 1.66(1.11,2.50) | 0.02 | 1.21(0.76,1.92) | 0.42 | 1.54(0.96,2.45) | 0.07 | 1.57(0.98,2.50) | 0.06 |
| P for trend |  | <0.001 |  | 0.21 |  | 0.02 |  | 0.02 |
| **Chronic diarrhea** |  | | | | | | | |
| Low | ref |  | ref |  | ref |  | ref |  |
| Moderate | 0.78(0.56,1.08) | 0.13 | 0.78(0.56,1.09) | 0.15 | 0.81(0.58,1.14) | 0.22 | 0.82(0.59,1.14) | 0.22 |
| High | 0.51(0.37,0.70) | <0.001 | 0.53(0.37,0.77) | 0.001 | 0.58(0.40,0.84) | 0.01 | 0.58(0.40,0.84) | 0.01 |
| P for trend |  | <0.0001 |  | <0.001 |  | 0.003 |  | 0.003 |
| **Fecal incontinence** |  | | | | | | | |
| Low | ref |  | ref |  | ref |  | ref |  |
| Moderate | 0.91(0.57,1.44) | 0.67 | 1.09(0.68,1.74) | 0.71 | 1.13(0.72,1.78) | 0.58 | 1.14(0.73,1.80) | 0.55 |
| High | 0.62(0.41,0.94) | 0.03 | 1.04(0.67,1.61) | 0.85 | 1.11(0.71,1.75) | 0.63 | 1.12(0.72,1.75) | 0.61 |
| P for trend |  | 0.01 |  | 0.95 |  | 0.71 |  | 0.7 |

**LE8 classification**

Crude model: Unadjusted model

model 1: Adjusted for age and sex

model 2: Additionally adjusted for marital status, educational level, family size, annual family income, alcohol, and PHQ-9

**Health behaviors classification**

Crude model: Unadjusted model

model 1: Adjusted for age and sex

model 2: Additionally adjusted for marital status, educational level, family size, annual family income, alcohol, and PHQ-9

model 3: Additionally adjusted for, BMI, DM, Hypertension, Hyperlipidemia

**Health factors classification**

Crude model: Unadjusted model

model 1: Adjusted for age and sex

model 2: Additionally adjusted for marital status, educational level, family size, annual family income, alcohol, and PHQ-9

model 3: Additionally adjusted for vigorous physical activity and smoke

CVH: cardiovascular health; LE8: life’s essential 8; OR: odds ratios; CI: confidence interval; BMI: Body Mass Index; DM: Diabetes mellitus;

Low was defined as a score of 0 to 49, moderate of 50–79, and high of 80–100
